# Precise intracellular uptake and endosomal release of diverse functional mRNA payloads via glutathione-responsive nanogels

**DOI:** 10.1016/j.mtbio.2024.101425

**Published:** 2024-12-29

**Authors:** Rupali Dabas, Naveenan Navaratnam, Haruki Iino, Saidbakhrom Saidjalolov, Stefan Matile, David Carling, David S. Rueda, Nazila Kamaly

**Affiliations:** a Cellular Stress Research Group, MRC Laboratory of Medical Sciences, Imperial College London, W12 0HS, London, UK; b Department of Chemistry, Molecular Sciences Research Hub, Imperial College London, W12 0BZ, London, UK; c Single Molecule Imaging Group, MRC Laboratory of Medical Sciences, Imperial College London, W12 0HS, London, UK; d Section of Virology, Department of Infectious Disease, Imperial College London, W12 0HS, London, UK; e Department of Organic Chemistry, University of Geneva, Geneva, Switzerland

**Keywords:** Nanogels, Glutathione-responsive nanogels, disulphide crosslinker, mRNA delivery, AMPK, GFP mRNA, Mango II RNA aptamers

## Abstract

We present a novel, highly customizable glutathione-responsive nanogel (NG) platform for efficient mRNA delivery with precise mRNA payload release control. Optimization of various cationic monomers, including newly synthesized cationic polyarginine, polyhistidine, and acrylated guanidine monomers, allowed fine-tuning of NG properties for mRNA binding. By incorporating a poly(ethylene) glycol-based disulphide crosslinker, we achieved glutathione-triggered mRNA release, enabling targeted intracellular delivery. Our NGs demonstrated superior encapsulation (up to 89.3 %) and loading (10.7 %) efficiencies, with controlled mRNA release kinetics at intracellular glutathione concentrations. NGs outperformed commercial transfection reagents across multiple cell lines, including traditionally difficult-to-transfect lines. We demonstrate the platform's versatility by successfully delivering GFP mRNA, Mango II RNA aptamers, and functionally relevant β2-AMPK mRNA. Furthermore, we used TIRF microscopy to measure exact RNA copy number within the NGs. Notably, mechanistic cellular uptake studies revealed that disulphide-containing NGs exhibit enhanced cellular uptake and endosomal escape, potentially due to interactions with cell surface thiols. This work represents a highly tuneable, efficient, and biocompatible platform for mRNA delivery with relevance for gene therapy and vaccine development.

## Highlights

• A novel glutathione-responsive nanogel (NG) platform enables efficient and controlled mRNA delivery.
• Novel cationic monomers provide optimized, precise mRNA binding.
• NGs demonstrate nearly 90% encapsulation efficiency and >10% loading efficiency, with glutathione-responsive release.
• NGs outperform commercial transfection reagents in traditionally difficult-to-transfect cell lines.
• Platform exhibits enhanced cellular uptake and endosomal escape, achieving functional protein translation.

## Graphical abstract

**Figure:**
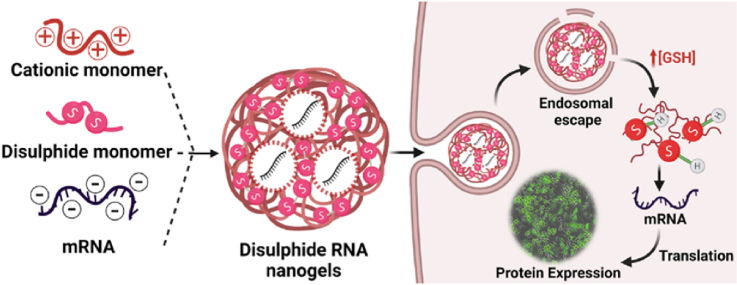

## 1 Introduction

In recent years mRNA-based therapeutics have gained considerable interest in the treatment of infectious diseases, cancers and genetic disorders [1,2]. RNA-based nanotherapeutics have transformed the biomedical research landscape with their tremendous potential for highly specific disease intervention at the genetic level, including the highly successful recent COVID-19 mRNA lipid nanoparticle (LNP) based vaccines [[2], [3], [4], [5], [6]]. However, when delivered *in vivo*, mRNA in its naked form exhibits a short half-life (0.25–2 h) in plasma due to its susceptibility to nuclease-mediated degradation, resulting in limited cell internalization and therapeutic potential [7,8]. Therefore, there is great interest in the development of safe and efficient vectors for mRNA delivery. To date, this field has been largely dominated by lipid nanoparticles (LNP) based systems. However, the design of LNPs offers limited flexibility, for instance, in mediating controlled release of nucleic acid payloads. Furthermore, cationic lipid-based formulations have been reported to induce toxicity and undesirable side effects, rendering them not amenable to frequent dosing as a result, limiting their dose and frequency of use [9,10]. Consequently, there is a need for rationally designed nanoplatforms to safely and effectively deliver therapeutically viable mRNA payloads. Here, robust covalent delivery vehicles, in particular, cross-linked and co-polymerized polymeric nanogels (NGs) demonstrate considerable chemical versatility, resulting from the ability to conjugate multiple functional groups onto the same polymer backbone, which can be covalently crosslinked and therefore, minimize premature diffusion or release of the mRNA payload until a stimulus is met [11,12]. NGs are effectively nanoscale hydrogels, that possess extensive cargo-loading capacities, low toxicity and exceptional biocompatibility [13]. Their unique chemical flexibility enables the rational improvement of NGs using a modular design, which can be adjusted by altering the co-monomer type, ratio and crosslinker density, permitting precise control over their functionality, morphological characteristics, payload release profiles, mechanical strength and biocompatibility [14].

To date, NGs have demonstrated effective delivery of a variety of RNA payloads [[15], [16], [17], [18], [19], [20]]. Furthermore, by installing moieties that respond to the physical and chemical stimuli in biological environments [[21], [22], [23]], multifunctional NGs can be developed to improve biological specificity through the precise spatiotemporal control of RNA delivery [24]. Biochemical stimuli include pH [25], temperature [26], enzymatic activities [27] and redox reactions [28]. Among these, the redox gradient between the intracellular and extracellular environments is the most extensively exploited. The antioxidant glutathione (GSH) controls extracellular and intracellular redox potentials, where its concentration ranges between 2 and 20 μM and 1–10 mM, respectively [29,30]. Therefore, GSH-mediated reduction of disulphide bonds has been explored for cytosolic delivery. Indeed, disulphide bonds have been installed within polymeric nanomaterials by using disulphide-containing crosslinkers [31,32] and by the self-assembly of biodegradable polymers from disulphide-containing monomers [33], making this a promising strategy for mediating spatial control over RNA delivery [34,35]. Furthermore, it was recently proposed that disulphide-bearing nanoparticles can facilitate rapid cellular uptake, while enabling rapid endosomal escape due to interactions with exofacial thiols on the cell membrane surface [36].

We have previously developed disulphide cross-linked nanogels for protein delivery [37] and in this study we aimed to exploit the intracellular redox gradient as a verified strategy to facilitate controlled intracellular mRNA release from NGs that incorporate a range of known and newly synthesized cationic moieties. Indeed, by the installation of disulphide crosslinkers, NGs can be engineered to degrade at cytosolic GSH concentrations (1–10 mM) that are substantially higher than in the extracellular environment (∼2–20 μM) [38,39]. Our NG design criteria aimed to create: (1) uniform particles with a diameter below 200 nm and polydispersity index (PI) under 0.2, (2) suitable effective surface charge (|zeta potential| > 30 mV) to enable cellular internalization and serum stability [40], (3) high RNA entrapment efficiency, and (4) intracellular release of functional RNA in response to cytosolic GSH.

Once we optimized the mRNA complexation ability of our NG platform, we investigated the subsequent transfection efficiency with mRNA encoding green fluorescent protein (GFP). We compared the transfection efficiency of our platform to Polyplus® jetMESSENGER, a polymer-based transfection reagent, that aligns more closely with our platform than lipid-based systems. Nonetheless, to offer a more comprehensive insight into NG transfection efficiency we have also included a comparison to the liposomal reagent Lipofectamine™ MessengerMAX™. To extend the application scope of our platform, we encapsulated fluorogenic RNA aptamers. These are typically used as biosensors for tracking the subcellular localization of RNA [41,42]. Of the wide variety of RNA aptamers, Mango II RNA (MII-RNA) aptamers have been discovered to possess a strong affinity and excellent fluorescent properties when bound to the fluorogenic TO1-Biotin [[43], [44], [45]]. We used this in combination with total internal reflection fluorescence (TIRF) microscopy to precisely estimate the number of RNA molecules in each nanogel. To further establish functional expression, we investigated the delivery of the biologically relevant mRNA encoding the β2 subunit of AMP-activated protein kinase (AMPK). AMPK is a key regulator of systemic energy homeostasis through its coordinated effects on the central nervous system and peripheral tissues [[46], [47], [48], [49]]. Loss of AMPK activity leads to metabolic dysfunction as well as defects in growth and development. AMPK is a multi-substrate, heterotrimeric serine/threonine kinase comprising of one α, one β, and one γ subunit [49]. In particular, the β subunit of AMPK regulates the phosphorylation status and activity of the AMPK complex. Two isoforms of the β subunit have been described, namely, β1 and β2, where β1 is primarily expressed in the liver, pancreas, kidney, brown fat and brain, and β2 is predominantly expressed in cardiac and skeletal muscle [50]. By demonstrating the effective and sustained delivery of various RNA payloads across a plethora of cell lines, we demonstrate the superior capability of our nanogels in comparison to industry standards. In doing so, we present our cationic, disulphide-crosslinked NGs as a complementary delivery platform to LNPs, specifically for therapeutic applications requiring precise, spatiotemporal control over RNA release.

## 2 Results and discussion

**Fig. 1:**
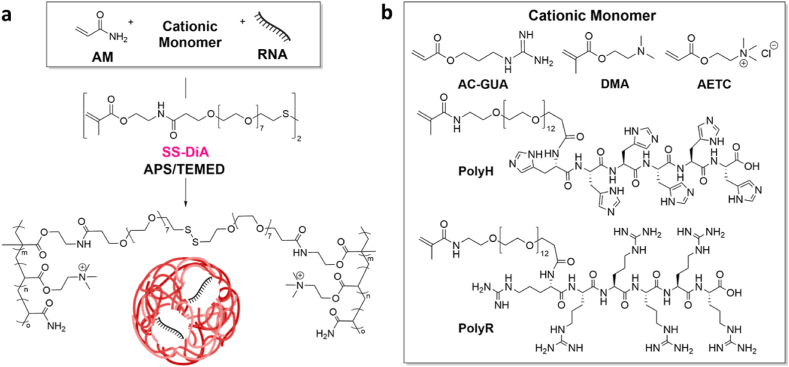
Synthesis scheme of a) disulphide-bearing nanogels for enhanced mRNA delivery and constituents and b) cationic/protonatable monomers screened for mRNA encapsulation within nanogels.

**NG design optimization: screening monomers for mRNA loading efficiency.** In our previous work, we had synthesized the disulphide NG platform using a free radical polymerization technique for protein delivery [37,51]. In this work, we employed the same disulphide-based crosslinker to confer glutathione-responsiveness to the NGs, (Supporting Information, Figs. S1a and b), but further investigated and screened a range of cationic commercially available and in-house synthesized monomers for the *in situ* encapsulation of the mRNA payload (Fig. 1a).

First, the reaction was optimized to prevent RNA degradation by minimizing the synthesis duration whilst achieving optimal NGs for enhanced cellular uptake. All NG formulations were prepared in nuclease-free water following a previously optimized synthetic protocol [37]. The crosslinker conversions and reaction completion times were investigated by varying initiator concentrations (Fig. S2a). Conversions were determined by measuring the vinyl olefin signals in the respective ^1^H NMR spectra (using sodium acetate as an internal standard) and NG size was measured using dynamic light scattering (DLS) (Figs. S2b and c) [52]. Notably, higher ammonium persulphate (APS) initiator content increased the crosslinker conversion and reduced the reaction time, but led to the formation of larger NGs (∼500–700 nm, Fig. S2a). Faster polymerization kinetics can result in uncontrolled radical coupling, thereby relinquishing control over particle size [53]. Therefore, 15 mol% of APS was used as it provided modest monomer conversions of 71 % and 30 min reaction time with NG sizes of 147.6 ± 50.0 nm and PI of 0.030 ± 0.001 (Fig. S2a). The NG was lyophilized and subjected to ^1^H NMR and FT-IR analysis to confirm the incorporation of the crosslinker (Fig. S3).

To further optimize the structural characteristics and mRNA complexation efficiency of our platform, we tested five different cationic/ionizable monomers (Fig. 1b). While the permanent cationic charge offered by 2-(acryloyloxy)ethyl trimethylammonium chloride (AETC) could permit electrostatic interactions with the RNA, excessive cationic charge has reportedly resulted in cytotoxicity [54]. As such, we investigated the use of (2-dimethyl)aminoethylmethacrylate (DMA) as an ionizable monomer alongside AETC to circumvent any potential cytotoxicity. DMA has amine groups capable of protonation at physiological pH and as a result, can interact electrostatically with negatively charged nucleic acids [55]. Further, the ionizable nature of DMA could enhance endosomal escape of NGs via the proton sponge effect [55].

While AETC and DMA were commercially available, the monomers polyarginine (PolyR), polyhistidine (PolyH) and acrylated guanidine (AC-GUA) were designed and synthesized. The design of PolyR and PolyH was inspired by cell-penetrating peptides (Figs. S4a and b, respectively), and they were synthesized using solid-phase peptide synthesis and characterized using ^1^HNMR (Figs. S5 and S7) and matrix assisted laser desorption ionization-time of flight mass spectrometry (MALDI-TOF MS) (Figs. S6 and S8) [[56], [57], [58], [59]].

Where in PolyR, we made use of the guanidinium motif due to its ability to interact electrostatically with the negatively charged RNA at neutral pH, in PolyH the imidazole rings permits hydrogen bond interactions with the RNA [[60], [61], [62]]. Both the polyarginine and polyhistidine have also demonstrated endosomal escape capacities [59]. Both peptides were coupled to a bifunctional PEG and acrylated to permit covalent polymerization within the NGs. To our knowledge our study is the first of its kind to present the design and mRNA complexation abilities of these peptide-based monomers.

Lastly, the AC-GUA, or acrylated-guanidine monomer, was similarly inspired by ability of the guanidinium motif to form salt bridges with the RNA phosphodiester backbone (Fig. S9) [63,64]. AC-GUA was synthesized via a two-step reaction involving nucleophilic displacement of ethyl carbaminidithioate by the amine group in 3-aminopropan-1-ol, followed by esterification of the primary alcohol using acryloyl chloride and then characterised by ^1^HNMR (Fig. S10), atmospheric-pressure chemical ionization mass spectrometry (APCI-MS) (Fig. S11) and FT-IR (Fig. S12).

**Fig. 2:**
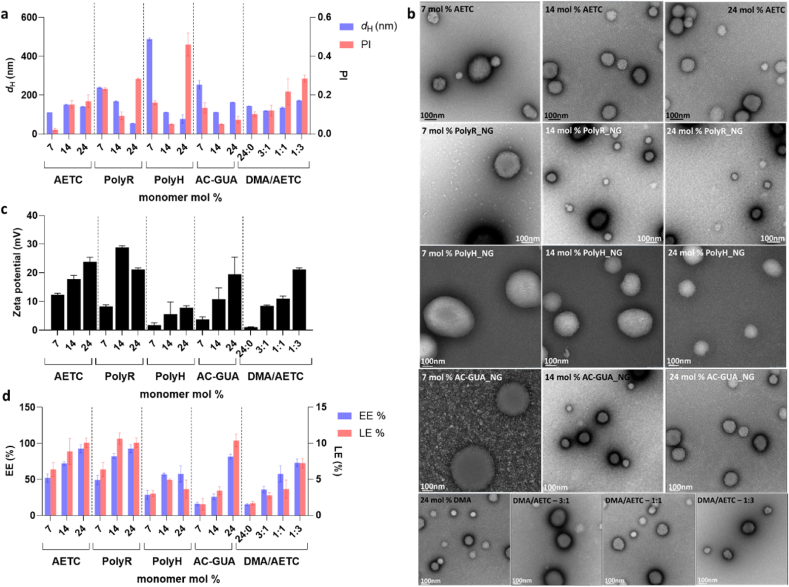
Characterization of polymeric NGs for mRNA delivery. a) Physicochemical characterization of size (hydrodynamic diameter) and polydispersity (PI) as obtained from dynamic light scattering measurements. For each figure the ratios displayed on the x axes relate to the mol % of the respective monomers incorporated within the NGs. (b) Negative stain transmission electron micrographs of NGs (45,000 x). Scale bar at 100 nm. (c) Zeta potential (mV) of synthesized NGs as obtained from electrophoretic scattering measurements (measured in water, pH 7.2). (d) Encapsulation and loading efficiency of mRNA within the NGs. In all cases, data are presented as mean ± standard deviation for n = 3.

As before, the monomer feed compositions were analysed using ^1^HNMR (representative example in Fig. S3) and the physicochemical properties were characterized using DLS, electrophoretic light scattering (ELS) and transmission electron microscopy (TEM) [37]. AETC_NG and PolyR_NG demonstrated desired sizes and PI at 24 mol % (141.1 nm, PI: 0.169) and 14 mol % (168.9 nm, PI: 0.094) of the respective monomers (Fig. 2a and b). These formulations also exhibited the most positive zeta potentials (23.9 ± 1.6 mV and 28.8 ± 0.6 mV, respectively) which is indicative of their colloidal stability (Fig. 2c). NG formulations consisting of 14 mol % PolyH, 24 mol % AC-GUA and 3:1 DMA/AETC, formed NGs that were <200 nm with PI < 0.2, and were considered to be morphologically optimal in their respective categories (Fig. 2a and b). However, they could not achieve comparative zeta potentials to AETC_NG and PolyR_NG, which could indicate compromised colloidal stability

and in turn, decreased RNA entrapment efficiency (Fig. 2c). Indeed, both 24 mol % AETC_NG and 14 mol % PolyR_NG exhibited highest RNA encapsulation (EE) and loading efficiencies (LE) (AETC: 92.7 %, 10.1 %; PolyR: 82.3 %, 10.7 %) (Fig. 2d). From these measurements, the congruency observed between the zeta potentials and the RNA EE/LE% indicates that RNA present within the NG core minimally affects the effective surface charge. This is further evidenced by the minimal changes in effective surface of the empty NGs for each monomer (Fig. S16).

In addition, the agarose gel measurements mirrored the EE/LE % analyses and highlighted minimal free RNA for 24 mol % AETC_NG (Fig. S13a). However, the other optimized formulations, namely 14 mol % PolyR, 14 mol % PolyH, 24 mol % AC-GUA and 3:1 DMA/AETC (henceforth known as AETC_NG, PolyR_NG, PolyH_NG, AC-GUA_NG and DMA/AETC_NG, respectively), exhibited slight RNA leakage as evident in their respective lanes (Figs. S13b,c,d,e). We then evaluated the RNA protective capacity of the optimized NGs by incubating with RNAse at 37 °C (Fig. S14). We observed that AETC_NG and PolyR_NG provided maximal protective benefits, whereas the other formulations exhibited slight RNA degradation. Taken together, these results indicate that AETC and PolyR were the most efficient at complexing and entrapping the RNA payload.

**Fig. 3:**
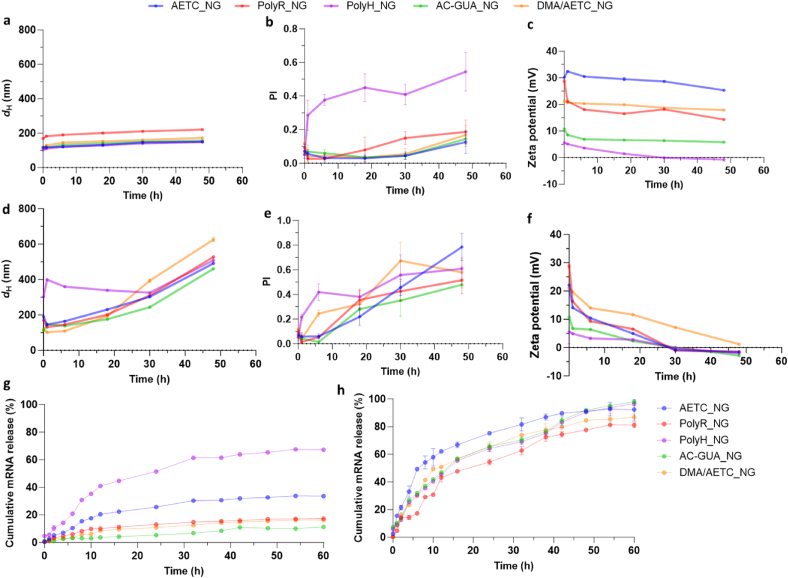
Characterization of NG structural changes upon exposure to GSH. Physicochemical characterization of NG a) size (hydrodynamic diameter), b) polydispersity (PI) and c) zeta potential measurements of NGs exposed to 20 μM GSH. Physicochemical characterisation of NG d) size (hydrodynamic diameter), e) polydispersity (PI) and f) zeta potential measurements of NGs exposed to 10 mM GSH. Cumulative RNA release from NGs incubated at 37 °C with [GSH]: g) 20 μM and h) 10 mM. In all cases, data are presented as mean ± standard deviation for n = 3.

**Assessment of redox-responsive behaviour of NGs.** In our previous work, we had characterised the release kinetics of protein payloads in the NGs in response to GSH [37]. In this work, we sought to determine whether changing the mRNA complexing monomer could influence the RNA release kinetics. To do so, we incubated the optimized NGs with GSH at extracellular (20 μM) and intracellular (10 mM) concentrations. As expected, all NGs demonstrated minimal size changes when left untreated or exposed to 20 μM GSH (Fig. 3a, Fig. S17). However, the PI for PolyH_NG increased dramatically in comparison to the other NGs, which indicates the instability of this formulation (Fig. 3b). Indeed, PolyH had the lowest zeta potential in comparison to other NGs, which decreased over the course of the experiment, as is expected for formulations with poor colloidal stability that are prone to aggregation (Fig. 3c) [65]. Interestingly, while PolyR_NG demonstrated minimal changes in size, the PI increased to 0.18 and the zeta potential decreased to ∼14 mV by 48 h which could imply some degradation of the NG. It is possible that the sterically hindering peptide motifs could prevent efficient packaging of the NG matrix. This then allows GSH to penetrate the NG structure, thereby triggering untimely degradation. AETC_NG demonstrated the best structural stability, with minimal changes in size, PI or zeta potential, highlighting the stability of this formulation. Upon treatment with 10 mM GSH, all NGs demonstrated controlled degradation over 48 h. Notably, all NGs, expect PolyH, decreased in size for up to 6 h post-incubation, which can be attributed to the rapid cleavage of the disulphide bonds at the surface of the NGs (Fig. 3d) [66]. This exposes the inner disulphide crosslinks to the surrounding environment, leading to an initial swelling and aggregation, as evidenced by the gradual increase in size and PI (Fig. 3d and e). These architectural changes were also confirmed by TEM (Fig. S15) We believe that due to the inherent instability of PolyH_NG, it continued to swell and aggregate leading to the structural changes observed (Fig. 3d and e). As expected, all NGs demonstrated dramatic decrease in zeta potential which was consistent with the degradation of the NG matrix (Fig. 3f).

To further confirm encapsulation stability, we measured the cumulative RNA release using the Quant-iT RNA quantification assay. At 20 μM, all NGs except PolyH_NG (67.2 %) demonstrated minimal RNA release, which was consistent with previously characterised structural instability of this formulation (Fig. 3g). While AETC_NG did demonstrate 22 % RNA leakage, it could be attributed to the release of any RNA molecules entrapped closer to the outer surface of the NGs. On the contrary, all NGs treated with 10 mM GSH demonstrated gradual, yet complete release of the encapsulated RNA over the course of the experiment, as expected (Fig. 3h). Interestingly, while AETC_NG exhibited the fastest RNA release (75.3 % within 24 h), PolyR_NG had the slowest (54.4 % within 24 h). This elucidates bioreducibility and specificity of our platform whilst highlighting the tunability of the NG system. Evidently, each monomer subtly modifies polymer architecture allowing us to expand and enhance the behavioural repertoire of these smart materials, thereby paving the way towards highly tailored nanoplatforms for therapeutic applications.

**Fig. 4:**
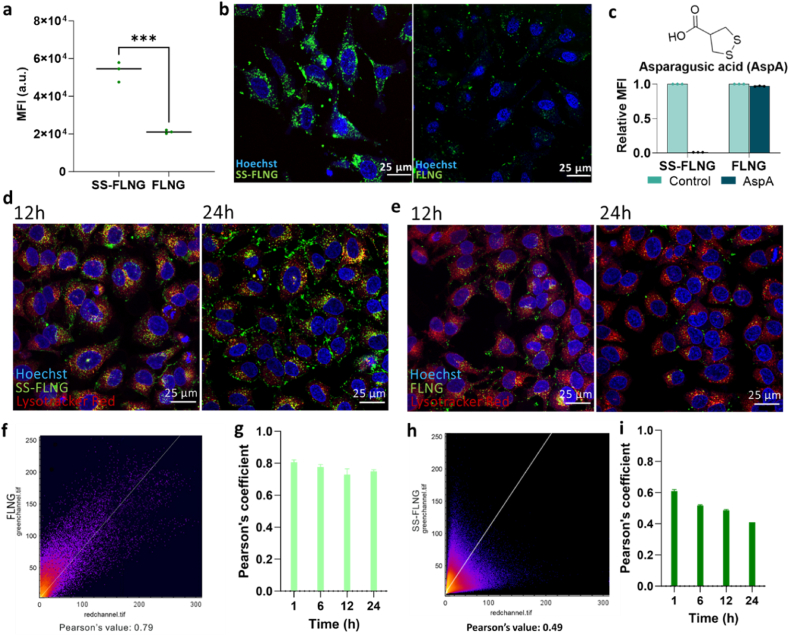
Cellular uptake and endosomal escape evaluation of fluorescent disulphide and non-disulphide NGs. a) Quantification of intracellular uptake of the two different NGs by flow cytometry. Mean fluorescence intensities (MFI %) of the two differently treated cell populations are shown (n = 6). Data shown are mean ± SD and statistical significance was determined using an unpaired *t*-test, ∗∗∗∗p < 0.0001. b) Confocal microscopy images showing the intracellular uptake of fluorescein-labelled disulphide (SS-FLNG) and non-disulphide NGs (FLNG) in HeLa cells fixed 8 h post-treatment, stained with 1 μg/mL Hoechst 33342. Images were taken with 63× oil immersion objective. Scale bar at 25 μm. c) Effect of the thiol mediated uptake inhibitor, asparagusic acid (AspA), on NG internalization (n = 3), as assessed by flow cytometry. Confocal microscopy images showing the intracellular uptake of d) disulphide (SS-FLNG) and e) non-disulphide (FLNG) fluorescent NGs at 12 h and 24 h. Cell stained with 75 nM Lysotracker Red and 1 μg/mL Hoechst 33342. All images were taken with 63× oil immersion objective. Scale bar at 25 μm. f) Pearson's correlation plot for FLNGs at 12 h and g) temporal evaluation of Pearson's coefficient for FLNGs; h) Pearson's correlation plot for SS-FLNGs at 12 h and i) the time dependent Pearson's coefficient evaluation.

**Evaluating cellular uptake and endosomal escape efficiency of disulphide NGs.** Disulphide-incorporating molecules and polymers have been recently shown to present enhanced cellular uptake and cytosolic presence, which has been attributed to a non-classical pathway involving surface-exposed thiols [36,[67], [68], [69]]. While single molecules can feasibly be uptaken through this non-classical pathway, larger nanoparticles are more likely to follow endosomal internalization pathways. Intrigued by this, we first studied the impact of disulphide moieties on both cellular entry and endosomal escape of the NGs. Although NGs are primarily internalized via the endosomal pathway, we find here that disulphide functionalities endow them with the ability for endosomal escape. Disulphide (SS-FLNG) and non-disulphide-crosslinked (FLNG) NGs were synthesized incorporating a fluorescently labelled monomer (fluorescein-*o*-acrylate, FL) to facilitate monitoring of cellular uptake (Fig. 4). Flow cytometry was used to compare cellular internalizations. Despite their similarity in size and zeta potential (SS-FLNG: 200.9 ± 2.9 nm and 22.1 ± 1.4 mV; FLNG: 219.7 ± 1.3 nm and 22.0 ± 1.3 mV) (Figure S18a-c,e), we observed a ∼2.5-fold increase in uptake in HeLa cells for the SS-FLNG, compared to the FLNG (Fig. 4a). This result was further confirmed by confocal microscopy (Fig. 4b, Fig. S19). We surmised that the higher uptake of the SS-FLNGs could be attributed to the interaction between disulphides in the NG structure and the cell surface thiols. To test this, we screened a library of exofacial thiol blockers, of which, asparagusic acid (AspA), was noted to be the most potent thiol-mediated uptake inhibitor (Fig. S20) [68,[70], [71], [72], [73]].

Indeed, we observed significant reduction in SS-FLNG uptake with AspA treatment, whereas FLNGs were unaffected (Fig. 4c). We then examined the relative propensity of the two NGs to undergo endosomal escape, by monitoring the colocalization of the fluorescent NGs with LysoTracker Red™ over 24 h, using confocal microscopy. SS-FLNGs demonstrated significant endosomal escape, as evidenced by the independent fluorescent colour from the LysoTracker Red™ and SS-FLNG (Fig. 4d). Indeed, even a few yellow, fluorescent foci, indicating endosomal entrapment, were resolved by 24 h. However, FLNGs were largely colocalized with Lysotracker even at the 24 h time point, indicating considerable endosomal entrapment (Fig. 4e). To quantify the extent of colocalization, we determined the Pearson's

coefficient. A high Pearson's coefficient number indicates colocalization, which makes the data points in the plot fall along the diagonal. Divergence of the points from the diagonal indicates disparate locations of the two fluorescent signals, corresponding to a lower Pearson's coefficient value [45]. Where the FLNGs exhibited a Pearson's value of 0.79 at 12 h (Fig. 4f,g, Fig. S21), a bifurcated distribution of fluorescent signals was observed for the SS-FLNGs by 12 h, giving a Pearson's value of 0.49 (Fig. 4h,i, Fig. S21). We did not observe any significant change in the Pearson's coefficient for FLNG over the entire 24 h time period, whereas, for the SS-FLNGs, the coefficient continuously decreased from an already lower 0.62 at 1 h to 0.41 by 24 h (Fig. 4i, Fig. S22). Despite the low Pearson's coefficient, the SS-FLNGs also exhibit punctate fluorescence in the cytosol, which can be attributed to endosomal entrapment [36]. This may be due to possible cytosolic aggregation of the NGs. As the fluorescein fluorophore is covalently conjugated within the NG framework, the formation of aggregates of degraded NGs can translate to localized, punctate fluorescence.

**Fig. 5:**
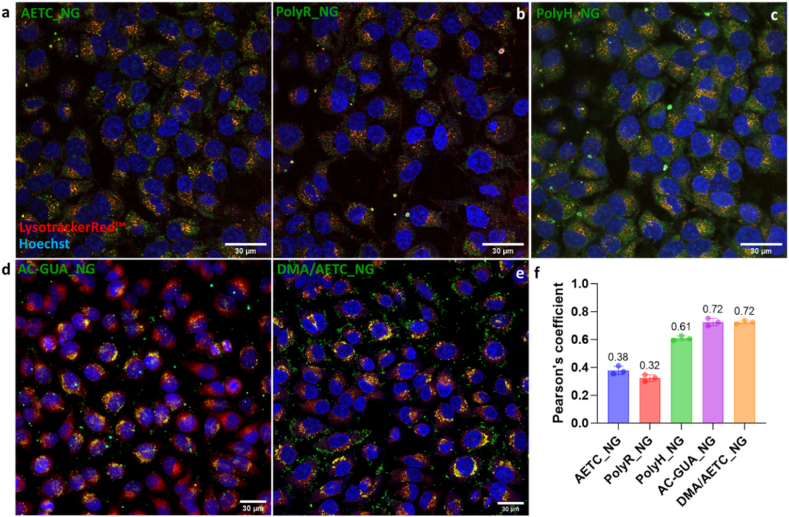
Evaluation of endosomal escape in HeLa cells. Confocal microscopy images showing the intracellular uptake of fluorescently labelled (green) a) AETC_NG, b) PolyR_NG, c) PolyH_NG, d) AC-GUA_NG and e) DMA/AETC_NG. Cells were stained 75 nM LysotrackerRed™ (red) and 1 μg/mL Hoechst 33342 (blue). All images were taken with 40× oil immersion objective. Scale bar at 30 μm. f) Pearson's correlation plot for all NGs.

We then sought to determine whether the incorporation of different cationic monomers in the optimized NGs, influenced their endosomal escape efficiency, a crucial factor for achieving successful intracellular RNA delivery. Indeed, the guanidinium moiety present in AC-GUA and PolyR can reportedly trigger endosomal escape via the proton sponge effect [64,74]. Further, the imidazole ring present in PolyH can augment endosomal lysis by osmotic swelling [75]. As before, we monitored the colocalization of fluorescently-labelled NGs with Lysotracker Red™ to assess endosomal escape. While the five types of NGs were similarly efficient at mediating cellular uptake, their endosomal escape capabilities differed considerably (Figs. S23a and b). As shown in Fig. 5, AETC_NG and PolyR_NG demonstrate significant endosomal escape, as evident by the independent fluorescent colour from the fluorescein-labelled NGs and Lysotracker Red™ (Fig. 5a,b, Fig. S23). On the contrary, PolyH_NG, AC-GUA_NG and DMA/AETC_NG were largely colocalized with Lysotracker Red™ indicating endosomal entrapment (Fig. 5c,d,e Fig. S23). To further quantify the extent of colocalization, we calculated the Pearson's coefficient for colocalization for all NGs (Fig. 5f). AETC_NG and PolyR_NG had Pearson's values of 0.38 and 0.32, respectively, whereas the other NGs had higher Pearson's values. Overall, these results highlight AETC_NG and PolyR_NG as the most promising candidates for mediating efficient endosomal escape, likely via the proton sponge effect facilitated by their cationic charge [[76], [77], [78]].

**Fig. 6:**
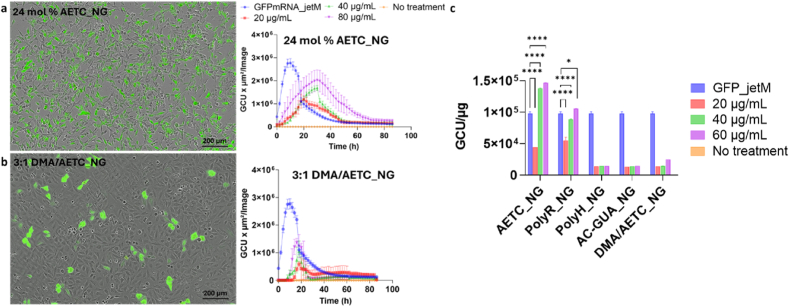
Representative *in vitro* NG GFP mRNA expression profiles with associated IncuCyte images of GFP expression in HeLa cells treated with 60 μg/mL (3.24 μg RNA) of NGs at 20 h for a) AETC_NG and b) DMA/AETC_NG. Each expression profile shows the fluorescence expression (green calibrated unit x μm^2^/image) as a function of time. In all cases, transfection efficiency was compared with the commercial reagent Polyplus® jetMESSENGER (GFPmRNA_jetM; 3.25 μg RNA). Scale bar for the images at 200 μm. c) Fluorescent protein expression normalized by total protein quantification (BCA assay, details in Methods section) for each NG dose, compared against untreated cells and cells treated with the commercial reagent Polyplus® jetMESSENGER®. Data analysed and evaluated for statistical significance by employing a two-way ANOVA with Tukey's multiple comparisons test (ns < 0.1234, ∗p < 0.0332, ∗∗p < 0.0021, ∗∗∗p < 0.0002, ∗∗∗∗p < 0.0001).

**High-throughput transfection efficiency assessment.** High-throughput screening was performed in 96-well plates by treating HeLa cells with the optimized NGs. We administered three different doses of the GFP mRNA-loaded NGs (20, 40 and 60 μg/mL; 0.55, 1.45, 3.24 μg RNA) and monitored GFP expression using the Incucyte® Live-Cell Analysis System for 86 h. JetMESSENGER® (jetM: 3.25 μg RNA) was used as a positive control following its demonstration of effective mRNA delivery in different cell models [79]. Fig. 6 and S24 show the fluorescence signal obtained per image for the different NG types with an associated image at 24 h. In all cases, NGs demonstrated concentration-dependent GFP expression, whereby higher NG concentrations increased the GFP signal, as observed. Notably, where the commercial reagent jetM enabled rapid transfection and maximum GFP expression within 12 h, NG-mediated transfection exhibited a gradual increase in GFP signal with maximal GFP expression ∼24 h and a more prolonged transfection profile.

This delayed and sustained protein expression was concordant with the controlled release profile of the NGs as observed *ex vivo*, highlighting the stimuli-responsiveness of the platform. Evidently, AETC_NG and PolyR_NG performed best, providing higher and more sustained GFP expression across all concentrations (Fig. 6a, Fig. S24a, respectively). While DMA/AETC_NG and PolyH_NG demonstrated similarly lower levels of transfection, AC-GUA_NG demonstrated minimal transfection (Fig. 6b, Figs. S24b and c, respectively). In absolute numbers, the commercial reagent provided higher and faster GFP expression prior to 24 h. However, after normalizing the fluorescence with total protein content, 40 and 60 μg/mL AETC_NG as well as 60 μg/mL PolyR_NG provided a significant increase in GFP expression compared to the commercial reagent (Fig. 6c). Although increasing the AETC_NG concentration from 20 to 40 μg/mL provided an ∼3 fold increase in GFP signal, any further increase in concentration did not provide a significant increase in GFP expression, indicating that the effective NG concentration should be around 40 μg/mL or higher. To then confirm the optimal concentration for *in vitro* delivery, we measured the cell viability of these formulations using the CCK-8 assay. Fig. S25 shows that treatment with AETC_NG, PolyR_NG and PolyH_NG upto 50 μg/mL did not cause any significant toxicity. However, due to the high cationic charge of these formulations, including AC-GUA, significant cytotoxicity was observed (∼55 % decrease) at concentrations higher than 250 μg/mL. Further, DMA/AETC_NG demonstrated lowest cytotoxicity among the NGs investigated presumably due to reduced cationic charge of the formulation.

**Fig. 7:**
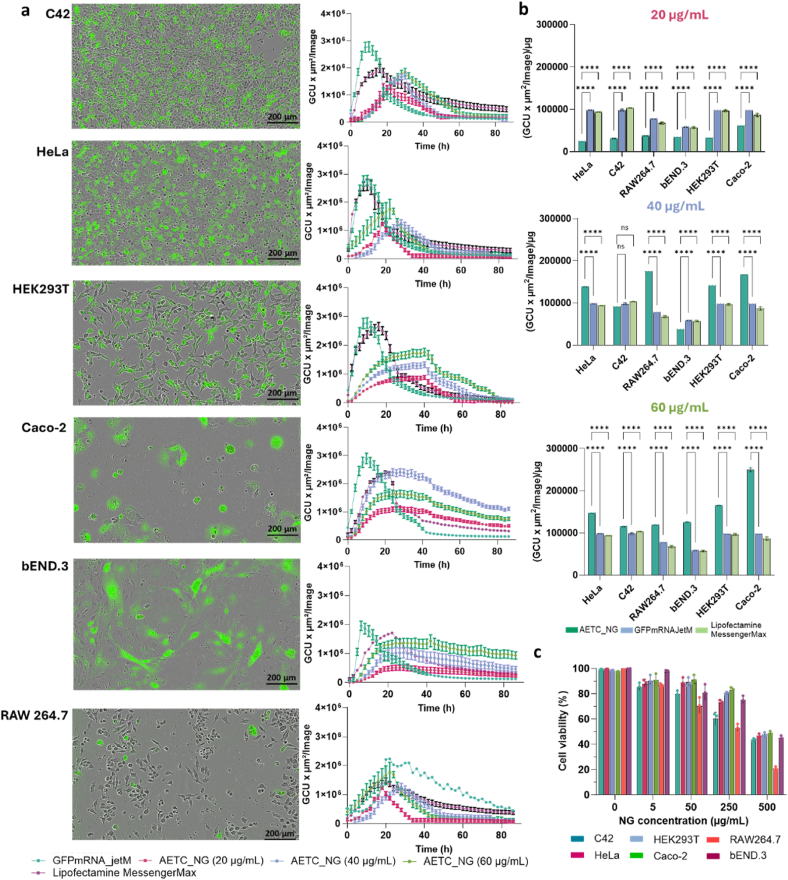
Comparing transfection efficiency of varying doses GFPmRNA_AETC_NG with Polyplus® jetMESSENGER® and LipoMessenger Max. a) The first panel shows representative Incucyte images of each cell line transfected with 60 μg/mL GFPmRNA_AETC_NG, at the 20 h time point. Scale bar is at 200 μm. The next panel shows the fluorescence expression (green calibrated unit x μm^2^/image) as a function of time for each cell line. b) Fluorescent protein expression normalized by total protein quantification (BCA assay, details in Methods section) for each mRNA_AETC_NG dose, compared against untreated cells and cells treated with the commercial reagent Polyplus® jetMESSENGER® and Lipofectamine™ MessengerMax™. Data analysed and evaluated for statistical significance by employing a a two-way ANOVA with Tukey's multiple comparisons test (ns < 0.1234, ∗p < 0.0332, ∗∗p < 0.0021, ∗∗∗p < 0.0002, ∗∗∗∗p < 0.0001). c) Cell viability determined for varying mRNA_AETC_NG concentrations in different cell lines. In all cases, data are presented as mean ± standard deviation for n = 3.

**Transfection Efficacy of AETC_NG in different cell lines.** AETC_NGs demonstrated improved transfection efficiency compared to other cationic nanogels, likely due to their superior cellular uptake and enhanced endosomal escape capabilities. However, as the efficiency of transfection can vary across different cell lines, owing to differences in uptake and intracellular transport mechanisms, the transfection efficiency of this formulation was evaluated in six different cell lines [[80], [81], [82]]. These included several “easy to transfect” (HEK293T, HeLa, Caco-2, C42) and “difficult to transfect” (bEND.3, RAW264.7) cell lines [83,84]. As before, three different doses of the GFP mRNA-loaded AETC_NGs (20, 40 and 60 μg/mL, containing 0.55 μg, 1.45 μg and 3.24 μg mRNA, respectively) were investigated. Fig. 7a shows the representative images and green fluorescence signal obtained per image of the different cell types. Data comparing transfection to untreated cells is included in Fig. S26. Representative images for the jetM transfected cells at the same time point are shown in Fig. S27a. Despite the variation in GFP expression across the different cell lines, AETC_NGs demonstrated a concentration-dependent increase in GFP expression, as expected (Fig. 7a). As before, the NGs demonstrated controlled release of the GFP mRNA, thereby enabling delayed and sustained GFP expression. While jetM and Lipofectamine™ MessengerMax™ displayed the highest and fastest GFP expression, when we normalized the fluorescence with total protein content, in order to account for different cell counts for the different cell lines, 40 and 60 μg/mL AETC_NG exhibited improved transfection efficiency compared to the commercial reagents (Fig. 7b). As anticipated, RAW264.7 and bEND.3 cells showed the lowest transfection efficiency [80,81]. Finally, the cytotoxicity of AETC_NG was evaluated in all six cell lines and was found to be minimal up to 250 μg/mL, whereas the commercial reagents demonstrated higher cytotoxicity in RAW264.7, Caco-2 and bEND.3 cells (Fig. 7c, Figs. S27b and c). Furthermore, the AETC_NGs demonstrated minimal haemolysis even at the highest concentration at 24 h, where only ∼19.28 % of RBCs were haemolysed upon incubation with 500 μg/mL NGs by 24 h (Fig. S28). Taken together, these results elucidate the biocompatibility and transfection potency of AETC_NG formulation whilst defining optimal concentrations to be in subsequent investigations.

**Fig. 8:**
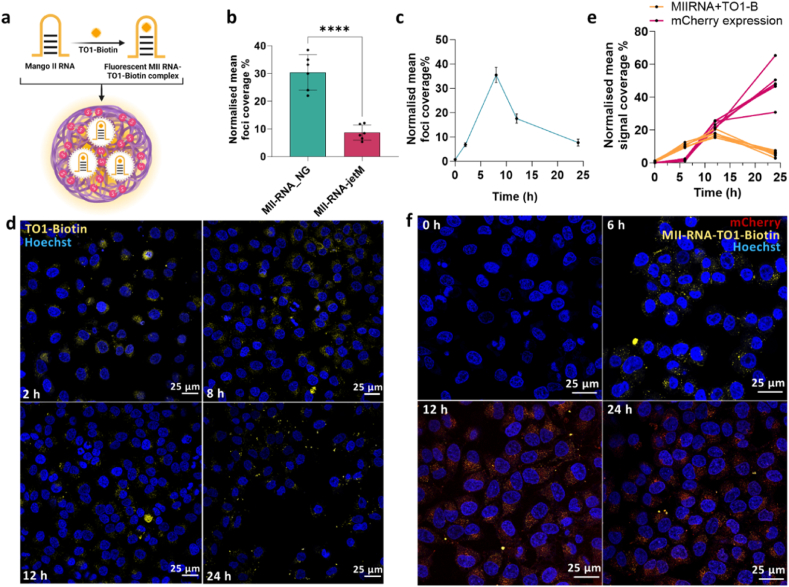
Evaluation of the transfection efficiency of MII-RNA-loaded NGs. a) Schematic presentation of TO1-biotin complexing with MII-RNA. b) Comparison of transfection efficiency against commercial reagent jetMESSENGER by determining Mango foci (n = 6). In all cases, data shown are mean ± SD and statistical significance determined using a one-way ANOVA with Tukey's multiple comparisons test, ∗∗∗p < 0.0002, ∗p < 0.0332, ns < 0.1234. c) Comparison of Mango foci signal coverage across different timepoints (n = 6). d) Confocal microscopy images of HeLa cells treated with MII-RNA_NGs and fixed at various time points. Cells were stained with 200 nM TO1-B (yellow) and 1 μg/mL Hoechst 33342 (blue). All images were taken with 40× oil immersion objective. e) Characterization of mChMII-RNA-loaded NGs and transfection - kinetics of Mango foci and mCherry signals (n = 6). f) Maximum projection of HeLa cells treated with mChMII-RNA-SS-NG, stained with 200 nM TO1-B (yellow) and 1 μg/mL Hoechst 33342. Images taken with 63× oil immersion objective.

**Evaluating transfection efficiency of AETC_NG using fluorogenic Mango II RNA aptamers.** To explore the potential for using our NGs as a tool for highly precise spatiotemporal cytoplasmic molecular detection of RNA, we decided to investigate the delivery of MII-RNA aptamers (Fig. 8a). The MII-RNA-loaded NGs mRNA (henceforth known as, MII-RNA_NG) demonstrated consistent size (102.3 ± 0.5 nm), PI (0.04 ± 0.04) and zeta potential (17.7 ± 0.4 mV) to NGs synthesized previously, which highlights the reproducibility of our synthetic strategy (Figs. S29a,b,c). Furthermore, the encapsulated RNA in our NGs demonstrated uncompromised fluorescence when compared to free RNA (Figure S29e). Since their complex three-dimensional structure, comprising of a three-tiered G-quadruplex [85], is critically conducive to the MII-RNA fluorescent properties, it was imperative to maintain their integrity upon encapsulation. Therefore, the NGs loaded with nine MII-repeat RNA (MIIx9-RNA) were subjected to photobleaching analysis under constant illumination using a TIRF microscope (Fig. 29e) [86]. A maximum likelihood estimate analysis of the photobleaching trajectory revealed that on average two 9x MII-RNA molecules (522 nucleotides) were entrapped in each NG. To evaluate the transfection efficiency, NGs were added to HeLa cells and compared against commercial reagents by counting the Mango II RNA foci (Image J FociPicker3D plugin). MII-RNA_NGs demonstrated a 3.5-fold increase in transfection efficiency relative to the commercial reagent (Fig. 8b, Fig. S30).

We also interrogated the kinetics of MII-RNA_NG uptake, noting that maximal RNA release occurred within 10 h, with maximal uptake (36 %) ∼8 h post-transfection followed by a gradual fluorescence decrease (Fig. 8c and d). This emphasises the role of the disulphide-based crosslinker in mediating the controlled release of the RNA payload from the NGs. Furthermore, the persistence of MII-RNA fluorescence, even at 24 h, highlights the NGs’ protective capacity.

Fluorogenic MII aptamers are an invaluable tool for visualising the subcellular localization of functional RNA transcripts [43,44]. Therefore, as a proof of concept, we loaded mRNA encoding for the mCherry protein tagged with 24 MII repeats within the disulphide-based NGs (mChMII-RNA_NG) [43]. The resulting NGs exhibited similar sizes to those synthesized previously, confirming the reproducibility of the synthesis (Figs. S31a–c). The LE (3 %) and EE (60 %) (Fig. S31b) were only marginally lower than those previously observed. This is likely due to the larger mRNA size (2097 nt). To test this, we loaded the disulphide-based NGs with varying sizes of MII-RNA transcripts. Despite having similar structural features to the NGs synthesized before, the observed EE% and LE% decrease with increasing RNA size, as expected (Figs. S32a–c). Further, we confirmed this result by TIRF-based photobleaching analysis to estimate the number of RNA transcripts within the NGs (Figs. S32d and e). To improve loading efficiency, it is possible to increase the amount of RNA added during NG synthesis in future studies.

Next, HeLa cells were transfected with mChMII-RNA-SS-NG formulation and the evolution of the MII-RNA and mCherry signal intensities was tracked over time using confocal microscopy (Fig. 8e,f, Fig. S33). While the RNA signal increases over the initial 12 h, in agreement with the previous results (Fig. 8e), the mCherry signal only increases 6 h post-transfection. This result is consistent with delayed RNA release from the NGs, followed by robust protein expression for >24 h. Indeed, the MII foci are clearly visible and independent of the diffused fluorescence expected from the mCherry protein expression, thereby further confirming that mCherry translation was not compromised by RNA encapsulation (Fig. 8f).

**Fig. 9:**
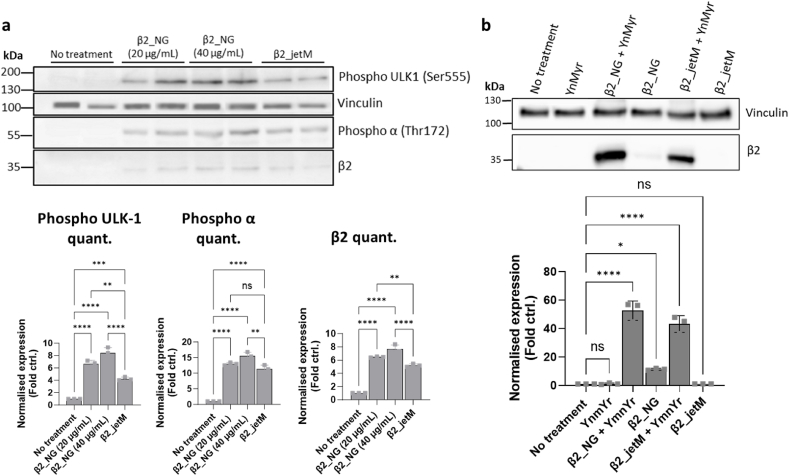
Western blots for cell lysates of HEK293T β2 knockout cells treated with β2 mRNA. A) Western blot confirming the overexpression of the β2 subunit and upregulation of phosphorylated AMPKα and ULK1. The graphs below display the quantification of the normalized band intensities and their comparison to untreated cells. B) Western blot confirming the post-translational modification (myristoylation) of flag-tagged β2 protein expressed by the delivered mRNA (∗YnMyr: tetradic-13-ynoic acid). The graph below displays the quantification of the normalized band intensities and their comparison to untreated cells. In all cases, data are presented as mean ± standard deviation for n = 3 and were evaluated for statistical significance by employing a one-way ANOVA, with Tukey's multiple comparisons test (ns < 0.1234, ∗p < 0.0332, ∗∗p < 0.0021, ∗∗∗p < 0.0002, ∗∗∗∗p < 0.0001).

**Evaluating transfection efficiency of AETC_NG using functional mRNA payloads *in vitro*: mRNA encoding β2 subunit of AMP-activated protein kinase.** With the initial successes in delivering our NGs to a variety of cell lines, we decided to investigate the delivery of an endogenously expressed, functionally relevant mRNA. We chose to investigate the delivery of mRNA encoding the β2 subunit of AMPK within the AETC_NGs in β2 knockout (KO) HEK293T cells. Given AMPK's significant role in cellular energy homeostasis, its potential as a therapeutic target has been extensively studied in the Carling lab, thereby providing a useful example for this proof-of-concept study [47,87,88]. We further studied its downstream target, unc-51 like autophagy activating kinase 1 (ULK1), to establish whether this protein would retain the same functions as endogenously expressed β2 mRNA. We hypothesized that the upregulation of the β2 subunit in this particular cell line would allow the formation of the total AMPK complex, thereby restore AMPK activity. This can be ascertained by increased phosphorylation at Thr172 (threonine 172) of AMPK and ULK1 [[89], [90], [91]]. To our knowledge, this study presents the first attempt to deliver β2 mRNA within our disulphide NGs. The β2 mRNA loaded AETC_NGs (henceforth known as, β2_NG) demonstrated consistent size (182.0 ± 2.1 nm), PI (0.030 ± 0.002) and zeta potential (22.0 ± 1.1 mV) to the ones synthesized previously, which highlights the reproducibility of our synthetic strategy (Fig. S34). We treated β2 KO cells with these nanoparticles for a period of 24 h and observed a significant increase in the expression of the β2 subunit in comparison to the untreated cells (∼7.7 fold) as well as in comparison to the cells treated with jetM (∼1.5 fold) (Fig. 9a). As seen for GFP mRNA, a dose dependent increase in β2 expression was observed. The increase in β2 expression was reflected in the increased phosphorylation of AMPK, as well as the increase in phosphorylation of ULK1, a downstream target of AMPK. These results indicate that the expressed protein retained its innate functionality.

To further confirm this, we investigated whether the expressed protein was able to undergo post-translational modifications that were crucial to its function. The β subunit isoforms of AMPK undergo constitutive amino-terminal myristolation, that is required for AMP- and ADP-triggered phosphorylation of Thr172 [87,92]. As such, we treated the HEK293T cells incubated with β2_NGs (40 μg/mL) and β2_jetM with a tagged myristate analogue, tetradic-13-ynoic acid (YnMyr), typically used to study myristoylated proteins due to its structural similarity to natural myristic acid [93]. We immunoprecipitated our flag-tagged β2 protein, which isolated protein expressed exclusively from our construct (Fig. 9b). We observed a ∼52.6-fold increase in the myristoylated protein isolated from β2_NG-treated cells in comparison to the untreated cells, and a ∼1.2-fold increase relative to β2_jetM-treated cells. Taken together, these results confirm that our nanoplatform is suitable for the delivery of RNA payloads encoding functionally complex endogenous proteins. In addition, our work demonstrates the post-translational modification of protein expressed by exogenously synthesized mRNA.

## 3 Conclusion

We presented the systematic optimization of NGs synthesized using various polymerizable monomers, including the newly synthesized monomers PolyR, PolyH and AC-GUA. Temporal examination of RNA transfection highlighted the controlled RNA release profiles of the NGs and their protective capacity as they maintained RNA integrity over a 24 h period. The varying release profiles exhibited by the monomers underscored the tunability of the NG platform, as specific structural features can be adjusted by altering the monomer type and ratio. This offers the unique potential to develop a precisely customized delivery platform to suit particular delivery applications. Furthermore, we presented methodical *in vitro* characterization of the five optimized NG formulations, of which AETC_NG was the most effective at mediating transfection. The enhanced efficiency of AETC_NGs can be attributed to their unique design that facilitates both efficient RNA complexation and enhanced cellular uptake. AETC functionalization imparts a positive surface charge, promoting electrostatic interactions with both the mRNA payload and the negatively charged cell membrane, leading to increased internalization, without compromising on cellular integrity. We further established the biocompatibility, transfection potency and expression longevity of these NGs across six different cell lines. As a further proof of concept, we first used MII-RNA to demonstrate the protective capacity of the NGs. We also used β2 mRNA to demonstrate that an exogenously synthesized mRNA can retain its innate roles and undergo relevant post-translational modifications upon delivery, thereby elucidating the protective encapsulation agency of the NGs. Using fluorescent NGs to study the mechanisms of cellular uptake and endosomal escape, we showed that disulphide NGs undergo faster endosomal escape than non-disulphide NGs. The temporal evolution of the endosomal escape supports the assertion that disulphide bonds play a critical role in the observed endosomal escape. However, the mechanism underlying endosomal escape still remains unclear. It is possible that the formation of covalent contacts between exposed thiols and disulphide NGs can strain the packing of the endosomal membranes, thereby leading to endosomal escape [36]. Elucidating this mechanism of endosomal escape can inform the design of better nanotherapeutic strategies for RNA delivery. Taken together, our results emphasise the versatility of covalent NG design for diverse delivery applications, achieved through customizable features such as stimuli-responsiveness, fluorescence, and the encapsulation of various RNA payloads. Finally, due to the ease of formulation preparation and consequent scalability of polymer synthesis, we anticipate that our NGs are highly clinically translatable as an RNA delivery platform.

## Materials and Methods

**Materials.** All reagents were from commercial sources and used as received. All chemicals, 2-aminoethyl methacrylatehydrochloride, triethylamine, methanol, anhydrous dichloromethane, 4,7,10,13,16,19,22,25,32,35,38,41,44,47,50,53-Hexadecaoxa-28,29-dithiahexapentacontanedioic acid di-*N*-succinimidyl ester, acrylamide, poly(ethylene) glycol diacrylate, glutathione, sodium dodecyl sulfate, ammonium persulphate, *N,N,N′,N′*-tetramethylethylenediamine were obtained from Sigma-Aldrich. All chemicals were used without further purification.

**Nanogel characterisation.**^1^H and ^13^C NMR spectra were recorded using a Bruker AV 400 MHz spectrometer at room temperature. Dynamic light scattering (DLS) was used to measure NG size, polydispersity index and zeta-potential using the Malvern Zetasizer Nano Ultra instrument with backscatter detection at 173° (n = 3). Each run measured 25 subscanning cycles. Prior to each measurement, the sample was filtered using MF-Millipore™ membrane filter (0.45 μm pore size, Merck Millipore, UK). The size measurements were taken at 25 °C for each NG synthesized. Zeta potential measurements were also conducted at 22 °C using a Zetasizer Nano-ZS instrument using a folded capillary cell in RNAse free water (pH 7.4). Transmission electron microscopy (TEM) samples 3.5 μL of NG suspension in water (1 mg/mL) was pipetted directly onto a glow-discharged lacey carbon film grid with 300 mesh copper (Agar Scientific, UK) and left on the grid for 1 min. Then the remainder of the solution was removed gently using a filter paper. The grid was then stained with 3.5 μL of 2 % (w/w) uranyl acetate for 1 min, after which the stain was removed gently using a filter paper. Samples were imaged on a Talos F200X G2 Transmission Electron Microscope at 45,000 x.

**Confirmation of cleavage of disulphide crosslinker with glutathione**. The redox-responsiveness of the synthesized crosslinker (4.55 mg/mL) was assessed by incubating the crosslinker with the reducing agent glutathione (GSH) at 20 mM for 24 h. MALDI-ToF (in ultrapure water) was used to detect the presence of the cleaved disulphide product. APCI-MS [M+H]^+^ (*m/z*): 577.7885, where M = CH_3_C(CH_2_)COOCH_2_CH_2_NHOOCCH_2_CH_2_O[CH_2_CH_2_O]_7_CH_2_CH_2_SH.

***In vitro* mRNA transcription.** mRNA encoding β2 was *in vitro* transcribed using respective DNA templates. In brief, the DNA templates for RNA Mango aptamers and β2 were produced by polymerase chain reaction (PCR) of the plasmid (mCherry-Mango II x 24 (plasmid #127587), Mango II x 9 plasmids, AMPK-β2-FLAG plasmid #40603, respectively) and adding T7 promoter sequence through PCR extension (BioMix™ Red, Bioline). After each PCR step, purification and gel extraction were performed (QIAquick Gel Extraction Kit, Qiagen), according to the manufacturer's instructions. mRNA was transcribed from the templates using the Invitrogen mMESSAGE mMACHINE™ T7 Transcription Kit, according to the manufacturer's instructions. The synthesized mRNA was then polyadenylated using the Invitrogen™ Poly(A) Tailing Kit, according to the manufacturer's instructions. mRNA was finally purified using the RNeasy Mini Kit (Qiagen), according to the manufacturer's instructions. The concentration and purity of the RNA was measured on a NanoDrop One (ThermoFisher, UK) and the RNA was stored at - 80 °C.

**RNA-loaded NG synthesis.** NG synthesis was conducted as described previously, with a few minor alterations to prevent RNA degradation [51]. Prior to the experiment, all equipment was UV-treated and oven-dried overnight to maintain RNAse-free reaction conditions. In addition, all surfaces were treated with RNAseZap™. RNAse-free water was used and was deoxygenated by nitrogen flushing overnight. Briefly, 6 μg of RNA was added to a 1 mL solution containing acrylamide (2 mg, 0.029 mmol, AM), disulphide crosslinker (4.5 mg, 0.004 mmol) and 2-[(acryloyloxy) ethyl] trimethylammonium chloride (AETC) or alternative cationic monomers (2 μL, 0.01 mmol) and this solution was left to stir for 30 min on ice under a continuous nitrogen atmosphere. For fluorescent NGs, fluorescein *o*-acrylate (0.2 mg, 0.005 mmol) was added to the monomer mix and the reaction was kept protected from light. Sodium dodecyl sulfate (8 mg, 0.028 mmol) was then added to enable micelle formation and left to stir for 30 min. Subsequently, to initiate free-radical polymerization ammonium persulfate (2.28 mg, 0.01 mmol) and *N,N,N′,N′-*tetramethylethylenediamine (2.5 μL, 0.022 mmol) were dissolved in 1 mL of RNAse free deoxygenated water and were added to the reaction flask. The reaction mixture was observed to achieve a pale blue opalescence within 30 min, which indicated reaction completion. To monitor monomer conversion, the reaction was conducted in deoxygenated D_2_O and aliquots were taken at predetermined time intervals for ^1^H NMR. The reaction was stopped by exposure to air once the characteristic opalescence was observed. The reaction mixture was then purified by centrifugation (1200×*g*, 10 min) using 15 mL 300 kDa MWCO Amicon® Ultra-15 centrifugal filter units (Sigma Aldrich, UK) with five washes against RNAse free water. The NGs were stored at 2–8 °C.

Molar ratio of monomers included in the study.

**Table:** 

**Monomer type**	**Molar quantity (mmol)**	**Molar ratio**	**Amount added**
Acrylamide	0.029	7.25	2.00 mg
AETC	0.010	2.50	2.00 μL
AC-GUA	0.010	2.50	1.72 μL
DMA	0.010	2.50	1.60 μL
PolyH	0.010	2.50	15.5 mg
PolyR	0.010	2.50	16.6 mg
Fluorescein o-acrylate	0.005	1.25	0.20 mg
PEG-disulfide acrylate	0.004	1.00	4.50 mg

**Encapsulation and loading efficiency measurements.** The encapsulation efficiency (EE %) of the RNA-loaded NGs was measured using the Quanti-It RiboGreen RNA assay kit (Invitrogen). In brief, after the NGs were synthesized, any external, uncomplexed RNA was quantified using this assay kit, according to the manufacturer's instructions. The encapsulation efficiency was further calculated using the following formula:

$$EE\;\%=\left(\frac{n\left({\text{RNA}}_{\text{total}}\right)-n\left({\text{RNA}}_{\text{external}}\right)}{n\left({\text{RNA}}_{\text{total}}\right)}\right)x\;100\%$$

To calculate the loading efficiency, the NGs were lyophilized and the following formula was used:

$$LE\;\%=\left(\frac{m\left({\text{RNA}}_{\text{loaded}}\right)}{m\left({\text{NG}}_{\text{empty}}\right)}\right)x\;100\%$$

**Characterisation of mRNA-NG complexation ability and mRNA stability by agarose gel electrophoresis.** The optimal AETC content to provide efficient mRNA encapsulation within the NGs was estimated using gel electrophoresis. Twenty microliters of the complex solution or 200 ng of naked mRNA as control was loaded on to Reliant™ Precast RNA Gels (1X MOPS buffer) and gel electrophoresis was performed. The gel was visualised using the iBright™ CL750 Imaging System (Invitrogen). To examine the stability of mRNA in the presecense of RNAse A, the formulation or naked mRNA was incubated wit RNAse A (Invitrogen) at 37 °C for 1 h, followed by mixing with RNA loaded dye (Thermo Fisher Scientific) and gel electrophoresis as described above.

**Quantification of RNA release from NGs in the presence of glutathione (GSH).** Disulphide and non-disulphide NGs were incubated with GSH at intracellular concentration (2–10 mM) at 37 °C. The mixture was allowed to react for predetermined time intervals at which aliquots were taken to determine the amounts of RNA released over time using the Quanti-It RiboGreen RNA assay kit (Invitrogen).

**Cell culture.** C42, bEND.3, RAW264.7, Caco-2 and HeLa cells were obtained from ATCC. β2-knockout HEK293Ts were prepared in-house at the Carling Lab [94]. C42 cells were cultured on poly-l-lysine (0.1 mg/mL, Thermo, UK) treated flasks and plates using RPMI 1640 medium (Thermo, UK), and supplemented with 10 % fetal bovine serum (FBS) and 1 % penicillin-streptomycin (Sigma-Aldrich, UK). bEND.3, RAW264.7, Caco-2 and HeLa cells were cultured in Dulbecco's Modified Eagle's medium (DMEM, GibcoTM) supplemented with 10 % FBS and 1 % penicillin-streptomycin (Sigma-Aldrich, UK). For all studies, the passage number for the C42, bEND.3 and RAW264.7 cells were kept below 25. All cells were maintained at 37 °C and 5 % CO_2_.

**Protocols for *in vitro* transfection studies (Mango RNA).** HeLa cells were seeded in Ibidi glass bottomed 8-well chamber slides (Ibidi, GmbH) at a seeding density of 50,000 cells/well and were left to adhere overnight prior to the transfection and imaging experiments. Mango RNA was transfected directly onto the cultured cells using RNA-loaded nanogels at various concentrations and for predetermined timepoints. Where relevant, the transfection efficiency of the nanoparticles was compared to that of PolyPlus jetMESSENGER® and Lipofectamine™ MessengerMax™, which were prepared according to the manufacturer's instructions. The commercial reagents were loaded with the same amount of RNA as was encapsulated within the nanogels to ensure consistency and improve validity of comparison.

**Cell fixing and preparation for imaging.** For imaging experiments with Mango RNA, the cells were fixed in ice cold PKM buffer (10 mM Sodium Phosphate, 100 mM KCl and 1 mM MgCl_2_) containing 4 % paraformaldehyde for 10 min on ice. Cells were then washed thrice with PKM buffer followed by a 20 min incubation with 200 nM TO1-B diluted in PKM buffer. The cells were washed thrice with PKM for 5 min each time. Then the cells were stained with Hoechst 33342 (1 μg/mL) in PKM buffer for 15 min. The cells were then washed thrice with PKM buffer and imaged on Leica TCS SP8 DLS inverted microscope (Leica, Germany) using LASX software (Leica, Germany). Images were analysed using ImageJ (NIH, USA).

**Photobleaching assisted microscopy using Total Internal Reflection Fluorescence (TIRF) microscopy.** The TO1-Biotin treated Mango RNA-loaded nanogels were loaded onto custom-made, quartz flow cells, passivated with PEG made by Dr Haruki Iino. For TIRF experiments, the constructs were immobilised on the surface via biotin-neutravidin interaction. The slide was washed twice with PKM buffer. Image acquisition for the Mango signal (488 nm laser) used 70 mW of power and 300 ms exposure time for 1000 frames. To determine the half-life of fluorescence, each photobleaching curve was fit to an exponential decay function. Maximum likelihood estimation was used to determine each of the photobleaching steps within a trace as previously described [86,95]. The step sizes were subsequently binned and the histogram was fit to a Gaussian equation.

**Image processing and quantification.** Images were processed by identifying the Mango foci by using the ImageJ plugin, Foci Picker 3D. Each foci detection algorithm was set to detect significant foci with a pixel area ≥ 3x3 pixels (≥100 nm diameter) and a peak intensity of ≥250 a.u (*n* = 6). To determine the foci coverage, the foci were first counted and then total cell area determined by outlining the cell boundaries using cell segmentation algorithm (CellProfiler). Then the area covered by the fluorescent signal was divided by the total cell area to obtain the percentage coverage.

**Protocols for *in vitro* transfection studies (GFP mRNA).** The C42, HEK293T, bEND.3, RAW264.7, Caco-2 and HeLa cell lines were seeded at a density of 1 × 10^4^ cells/well in 96 well cell culture plates in triplicate for each condition and left to adhere overnight prior to the experiment in the IncuCyte Instrument, placed in a normal humidified incubator at 37 °C with 5 % CO_2_. We treated the cells with varying concentrations of the GFP_AETC_NGs in culture media containing 10 % FBS. Where relevant, the transfection efficiency of the nanoparticles was compared to that of Polyplus® jetMESSENGER® and Lipofectamine™ MessengerMax™, which were prepared according to the manufacturer's instructions (prepared with 3.25 μg RNA). Automated phase contrast and green-fluorescence imaging was carried out. Scans were taken every 1 h with four pictures taken per well for 86 h (image resolution: 1.22 μm/pixel) to monitor expression of GFP for the desired time frame. Used default settings for image acquisition time in the green channel (800 msec). Data obtained was first optimized using the control, untreated cells to ensure we set a threshold for the background fluorescence. The IncuCyte software allowed adjustments in the processing definitions for analyses, so we optimized the confluence masks for the fluorescence and phase-contrast images. We adjusted the default settings to account for cell debris and to reduce the background fluorescence. Once a suitable setting was identified, this was used across all wells and all cell lines. Quantification of GFP fluorescence was automatically normalized to cell density in each well to account for any variability in cell number by the IncuCyte software. To quantify the total cell protein content, the cells were lysed in RIPA cell lysis buffer and centrifuged at 14,000 rpm for 10 min at 4 °C to isolate the protein. Then, the protein concentration was determined using the BCA protein assay (ThermoFisher, UK), according to the manufacturer's instructions.

**Endosomal escape studies using confocal microscopy.** 5 × 10^4^ HeLa cells/well were seeded in μ-Slide 8 Well chambered slides (Ibidi, GmbH) overnight. The next day, the media was replaced and the cells were incubated with 20 μg/mL of fluorescein-labelled NGs (FLNG and SS-FLNG) for 4 h. The cells were then washed thrice with PBS and treated with 75 nM Lysotracker Red for 30 min at 37 °C to stain the late endosomes/lysosomes. Then the cells were washed thrice with PBS and fixed with 4 % paraformaldehyde for 10 min on ice. The cells were then washed thrice and stained with Hoechst 33342 (100 ng/mL) in PBS for 15 min. The cells were then washed thrice with PBS and imaged using the Leica TCS SP8 DLS inverted microscope (Leica, Germany) using LASX software (Leica, Germany). Images were analysed using ImageJ (NIH, USA).

**Cellular uptake quantification studies.** 1 × 10^5^ HeLa cells/well were seeded in 24 well plate and incubated overnight. The cells were then washed with PBS and replaced with media to which 20 μg/mL of fluorescein-labelled disulphide and non-disulphide NGs were subsequently added. After 8 h of incubation, the media was removed and the cells were washed twice with PBS, trypsinised, and pelleted by centrifugation at 1200 rpm for 3 min. The supernatant was removed and the pellet was washed with 500 μL PBS and centrifuged under the same conditions. Then the PBS was removed and the cells were resuspended in FACS buffer (1 % BSA in PBS) and flow cytometry analysis was conducted using the FACSymphony™ A3 instrument. Data analysis was done using FlowJo software to calculate the mean fluorescence intensities (MFI %).

**Exofacial surface-thiol blocking study.** 1 × 10^5^ HeLa cells were seeded in a 12 well plate and incubated overnight. After 24 h of incubation, the media was replaced with the thiol-mediated uptake inhibitors (kindly provided by the Matile Lab, University of Geneva) at the following concentrations: DTNB (1.2 mM), IA (10 mM), AspA (500 μM), BI (10 μM), CTO (100 μM), EBS (100 μM), ETP (25 μM), MAC (100 μM), SS (10 μM) in DMEM for 1 h, at 37 °C. The working concentrations of the inhibitors were determined using values in previous publications and inhibitor solutions were prepared in culture media with <0.1 % final concentration of DMSO [[70], [71], [72], [73]]. Then the solution was removed, cells were washed with PBS (3 x 500 μL) and then, cells were treated with 50 μg/mL NG solutions in DMEM for 4 h (400 μL media). Finally, the cells were harvested following the same procedure as discussed above and analysed accordingly.

**Transfection efficiency quantification by western blots.** β2-knockout HEK293T and C42 cells: In brief, 1 × 10^6^ cells were seeded into 6 cm well plates (collagen coated) and incubated for 24 h. After adding fresh media, the plates were treated with various concentrations of the β2 or PGC1α-loaded NGs for predetermined time intervals. For protein collection, the cells were washed twice with ice cold PBS before lysis in RIPA buffer. Samples were then centrifuged at 14,000 rpm for 10 min at 4 °C to remove insoluble material. Protein content of the supernatant was quantified using a BCA assay kit (ThermoFisher, UK). Proteins were then resolved by NuPAGE™ 4–12 % Bis-Tris protein gels and transferred to polyvinylidene difluoride membranes. Primary antibodies were used at 1:1000 dilution. The primary antibodies used in this study as are follows: AMPKβ1β2 (Cell Signalling Technology, 4150), vinculin (Sigma Aldrich, V9131), phospho-AMPKα (Thr172) (Cell Signalling Technology, 2531), phospho-ULK1 (Ser555) (Cell Signaling Technology, D1H4) and anti-FLAG (Sigma Aldrich, F3165). Primary antibodies were detected using LI-COR IRDye Infrared Dye- (1:10,000 dilution) or HRP- conjugated secondary antibodies (1:3000 dilution) and visualised using an Odyssey Infrared Imager (LI-COR Biotechnology) or Amersham ImageQuant 800 system, respectively. Quantification of results were performed using Odyssey software (LI-COR) or ImageJ (ECL) and expressed as a ratio of the signal relative to the signal obtained using an appropriate control antibody.

**Cell cytotoxicity evaluation.** 1 × 10^4^ bEND.3, RAW264.7, Caco-2, HEK293T, C42 and HeLa cells/well were cultured in a 96 well plate and incubated overnight. Then, one concentration of jetMessenger® (0.25 μL – according to manufacturer's instructions for transfection in a 96-well plate) and various concentrations of the NGs (0–500 μg/mL) were investigated for 24 h and the cytotoxicity measured using the Cell Counting Kit-8 (Sigma Aldrich, UK) according the manufacturer's instructions.

**Haemolysis assay.** To determine impact of developed nanoparticles on cell membranes, the NGs were incubated with red blood cell (RBC) concentrate isolated from animal blood. 6 mL of whole blood sample was added to 12 mL PBS and then centrifuged at 3000 rpm for 10 min to isolate RBCs. The cells were further watched with 10 mL PBS until the supernatant was clear. Then, a 2 % RBC concentration was prepared by diluting the RBCs in sterile PBS (pH 7.4). Various concentrations of AETC_NGs (0–500 μg/mL) were incubated with 2 % RBC concentrate at 37 °C. In addition, 10 % SDS solution and PBS were used as positive and negative controls, respectively. After 2, 4, 8, 12 and 24 h, the samples were centrifuged at 2500 rpm for 5 min and 100 μL of the supernatant were transferred in a 96-well plate. The absorbance of released haemoglobin from erythrocytes was measured at a wavelength of 570 nm. Haemolysis percentages of the RBCs were calculated using the following formula:

$$\%\;Haemolysis=\frac{\left(abs\;of\;sample-abs\;of\;negative\;control\right)}{\left(abs\;of\;positive\;control-abs\;of\;negative\;control\right)}\times 100\;\%$$

Percent haemolysis values were calculated from three separate experiments.

**Statistical Analysis.** All statistical analyses were performed using GraphPad Prism. The *p* values were obtained by the Student's *t*-test, and the *p* value of <0.05 was considered statistically significant. The data were presented as mean ± standard deviation.

## CRediT authorship contribution statement

**Rupali Dabas:** Writing – review & editing, Writing – original draft, Validation, Methodology, Investigation, Formal analysis, Data curation. **Naveenan Navaratnam:** Writing – review & editing, Methodology, Formal analysis, Data curation. **Haruki Iino:** Writing – review & editing, Methodology, Formal analysis, Data curation. **Saidbakhrom Saidjalolov:** Writing – review & editing, Methodology, Formal analysis, Data curation. **Stefan Matile:** Writing – review & editing, Validation, Methodology, Formal analysis, Data curation. **David Carling:** Writing – review & editing, Validation, Supervision, Resources, Project administration, Methodology, Funding acquisition, Formal analysis, Conceptualization. **David S. Rueda:** Writing – review & editing, Validation, Supervision, Project administration, Methodology, Investigation, Formal analysis, Conceptualization. **Nazila Kamaly:** Writing – review & editing, Writing – original draft, Validation, Supervision, Resources, Project administration, Methodology, Investigation, Funding acquisition, Formal analysis, Conceptualization.

## Associated content

Schematic presentation of cationic monomer synthesis, ^1^HNMR spectra, FT-IR spectra, MALDI-ToF MS spectra, gel electrophoresis analyses, GFP mRNA expression profiles, cytotoxicity analysis, photobleaching curves for various Mango RNA payloads, complete physicochemical characterisation (DLS, ELS, TEM) of synthesized nanogels, confocal microscopy images of fluorescent nanogels, primers used for RNA synthesis.

## Notes

The authors declare no competing financial interest.

## Declaration of competing interest

The authors declare that they have no known competing financial interests or personal relationships that could have appeared to influence the work reported in this paper.

## Data Availability

Data will be made available on request.

## ABBREVIATIONS

AETC: [2-(Acryloyloxy)ethyl] trimethylammonium chloride
PolyR: polyarginine
PolyH: polyhistidine
AC-GUA: acrylated guanidine
DMA: 2-dimethyl)aminoethylmethacrylate
jetM: jetMESSENGER
DLS: dynamic light scattering
ELS: electrophoretic light scattering
TEM: transmission electron microscopy
PI: polydispersity
FT-IR: fourier-transform infrared radiation
NMR: nuclear magnetic resonance
MALDI-ToF: matrix assisted laser desorption ionization-time of flight mass spectrometry
APCI-MS: atmospheric-pressure chemical ionization mass spectrometry
AMPK: AMP-activated protein kinase
NG: nanogel
FL: fluorescein-*o*-acrylate
PEG: polyethylene glycol
GSH: glutathione
GFP: green fluorescent protein
PKM: (10 mM Sodium Phosphate, 100 mM KCl and 1 mM MgCl_2_)
SS-FLNG: disulphide fluorescent nanogel
FLNG: non-disulphide fluorescent nanogel
MII-RNA: Mango II RNA
