# Supplementary material for: Precise intracellular uptake and endosomal release of diverse functional mRNA payloads via glutathione-responsive nanogels
Source: Mater Today Bio. 2024 Dec 29;30:101425. doi: 10.1016/j.mtbio.2024.101425 (PMC11745970; doi:10.1016/j.mtbio.2024.101425)
Supplement: Multimedia component 1 [file mmc1.docx]

**Supporting Information for:**

Precise Intracellular Uptake and Endosomal Release of Diverse Functional mRNA Payloads via Glutathione-Responsive Nanogels

Rupali Dabas,^†,‡^ Naveenan Navaratnam,^†^ Haruki Iino,^§,¶^ Saidbakhrom Saidjalolov,^||^ Stefan Matile,^||^ David Carling,*^†^ David S. Rueda,^§,¶^* and Nazila Kamaly^‡^*

† Cellular Stress Research Group, MRC Laboratory of Medical Sciences, Imperial College London, W12 0HS, London, U.K.

‡ Department of Chemistry, Molecular Sciences Research Hub, Imperial College London, W12 0BZ London, U.K.

§ Single Molecule Imaging Group, MRC Laboratory of Medical Sciences, Imperial College London, W12 0HS, London, U.K.

¶ Section of Virology, Department of Infectious Disease, Imperial College London, W12 0HS London, U.K.

|| Department of Organic Chemistry, University of Geneva, Geneva, Switzerland

***To whom correspondence should be addressed:** [nazila.kamaly@imperial.ac.uk](mailto:nazila.kamaly@imperial.ac.uk), [david.carling@lms.mrc.ac.uk](mailto:david.carling@lms.mrc.ac.uk), [david.rueda@imperial.ac.uk](mailto:david.rueda@imperial.ac.uk)

Supplementary Figures


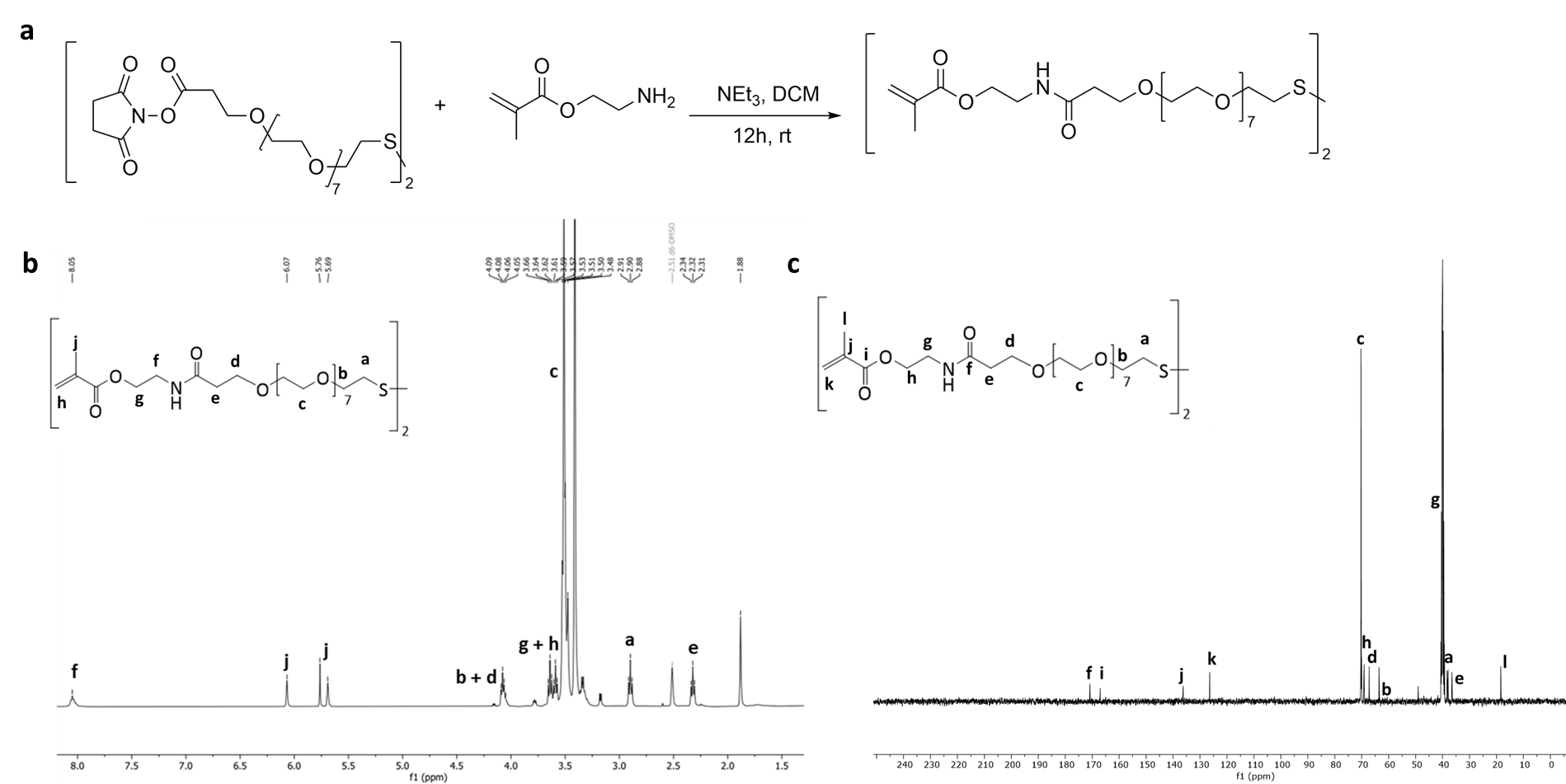


Figure S1. a) Synthesis of PEG disulphide diacrylate crosslinker. b) ^1^H NMR (400 MHz, (CD­_3_)_2_SO): δ8.05 (t, 2H), 6.07 (s,2H), 5.69-76 (m, 2H), 4.05-4.09 (m, 8H), 3.61-3.65 (t, 4H), 3.57-3.59 (t, 4H), 3.48-3.53 (m, 56H), 2.88-20.91 (t, 4H), 2.31-2.34 (t, 4H), 1.88 (s, 6H). *Residual solvents (CH_3_)_2_SO (δ 2.50); CH_2_Cl_2_(δ 5.76) c) ^13^C NMR (400 MHz, (CD_3_)_2_SO): δ171.04, 166.89, 135.18, 125.36, 69.98-70.24, 69.20, 64.38, 64.54, 38.23, 36.79, 35.58, 18.49.


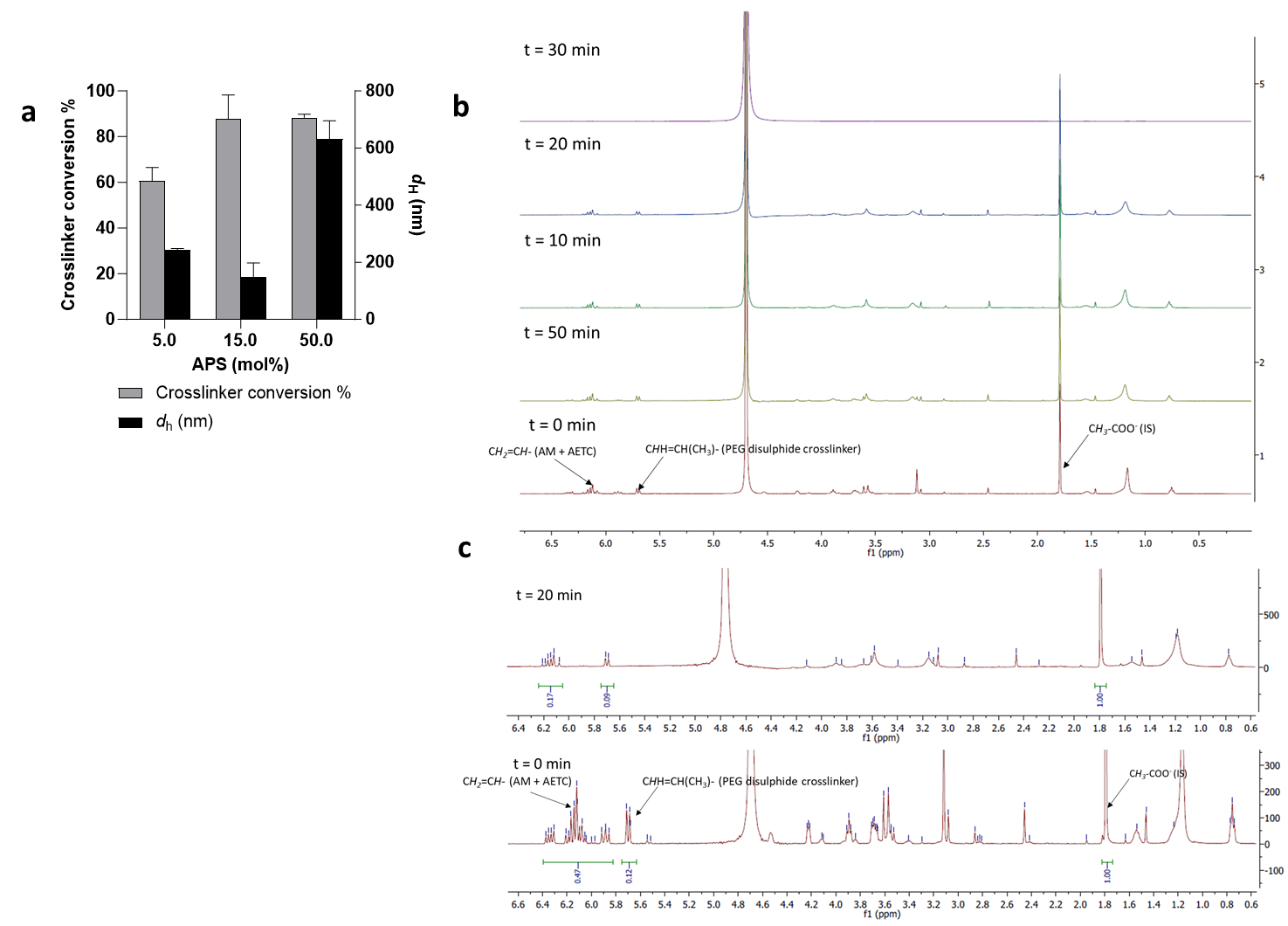
Figure S2. a) Graph comparing disulphide crosslinker conversion, as calculated by ^1^H NMR analyses, and nanogel size using various concentrations of initiator. Nanogels were polymerized with 5, 15 and 50 mol % of APS for 30 min. (n = 3) b) Reaction kinetics of polymerization monitored by ^1^H NMR by the gradual disappearance of the vinyl olefin signals. c) Partial ^1^H NMR spectra of the polymerization mixtures for the preparation of nanogels included in the study as a representative example. Peaks integrated to calculate the monomer conversions are indicated. All NMR were conducted in D_2_O. Sodium acetate was used as internal standard (IS). Intensities have been scaled for clarity.


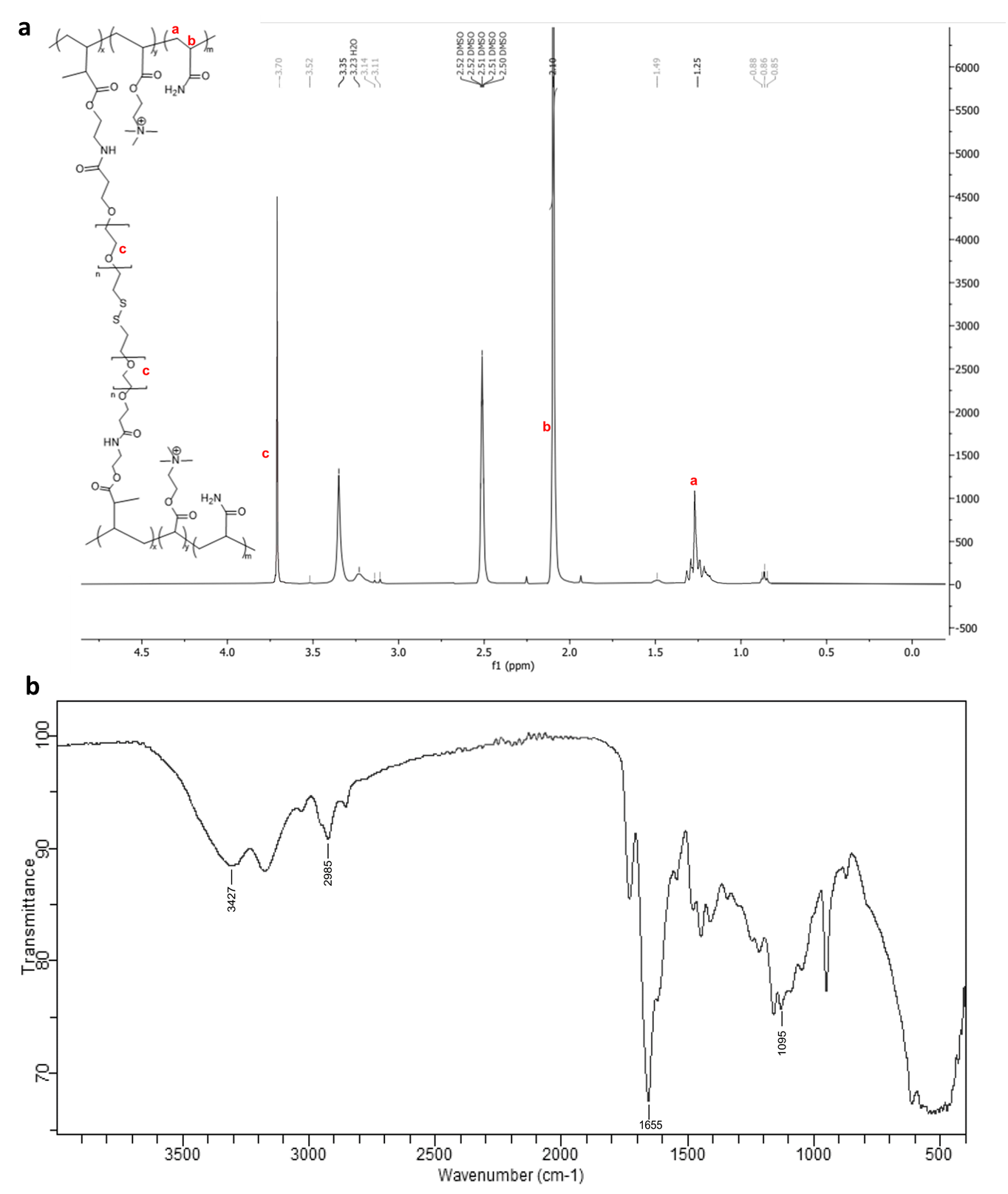
Figure S3. a) ^1^H NMR spectrum of lyophilized disulphide nanogels. ^1^H NMR (400 MHz, D_2_O): δ3.70 (s), δ2.1 (m), δ1.25 (m). b) Fourier transform infrared spectroscopy (FT-IR) of lyophilised nanogels. N-amine C(O)N-H stretch: 3700–3200 cm^-1^; C-H stretch: 2950–2850 cm^-1^ , C=O stretch: 1655 cm^-1^; C-O-C stretch: 1095 cm^-1^.


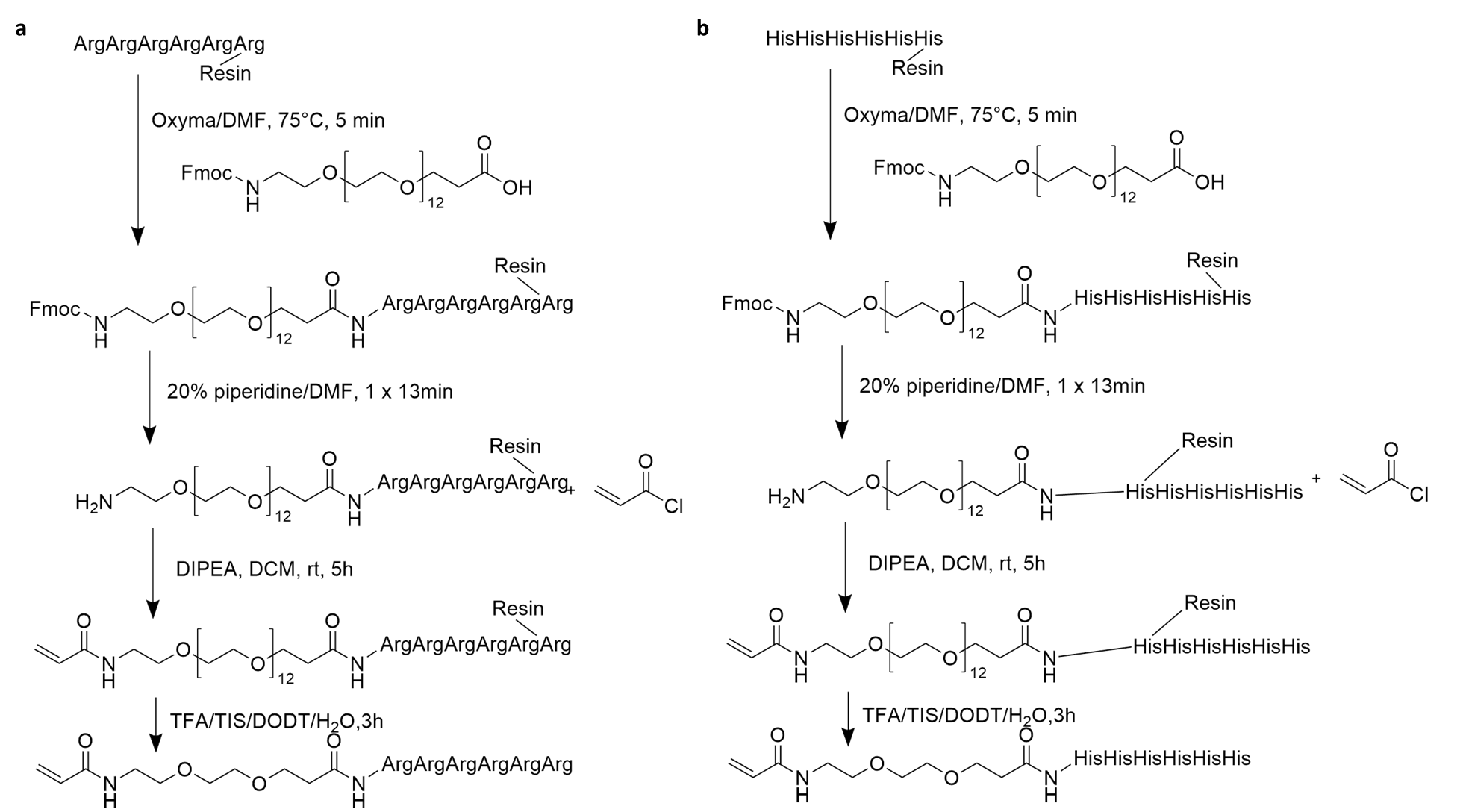
**Figure S4.** Synthetic scheme of a) PolyR and b) PolyH synthesis.


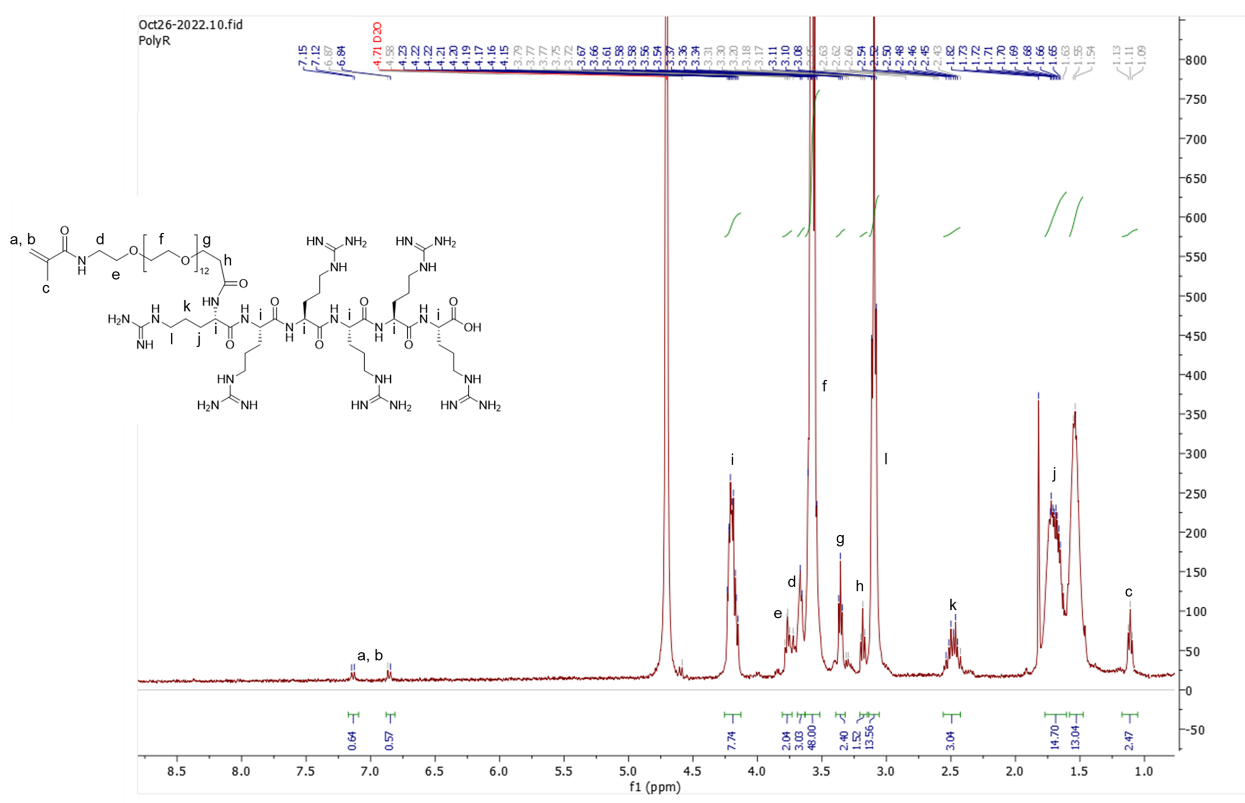
**Figure S5.** ^1^H NMR spectrum of PolyR. ^1^H NMR (400 MHz, D_2_O): δ7.15 (d), δ6.87 (d), δ4.23 (t), δ3.85 (t), δ3.77 (t), δ3.58 (tt), δ3.48 (t), δ3.34 (t), δ3.11 (t), δ2.50 (m), δ1.70 (q) δ1.11 (t).

^^**Figure S6.** Matrix assisted laser desorption ionization-time of flight – mass spectra (MALDI-ToF MS) of acrylated guanidine. M = (C_69_H_135_N_25_O_22_) [M + H]^+^ = 1666.9 m/z.


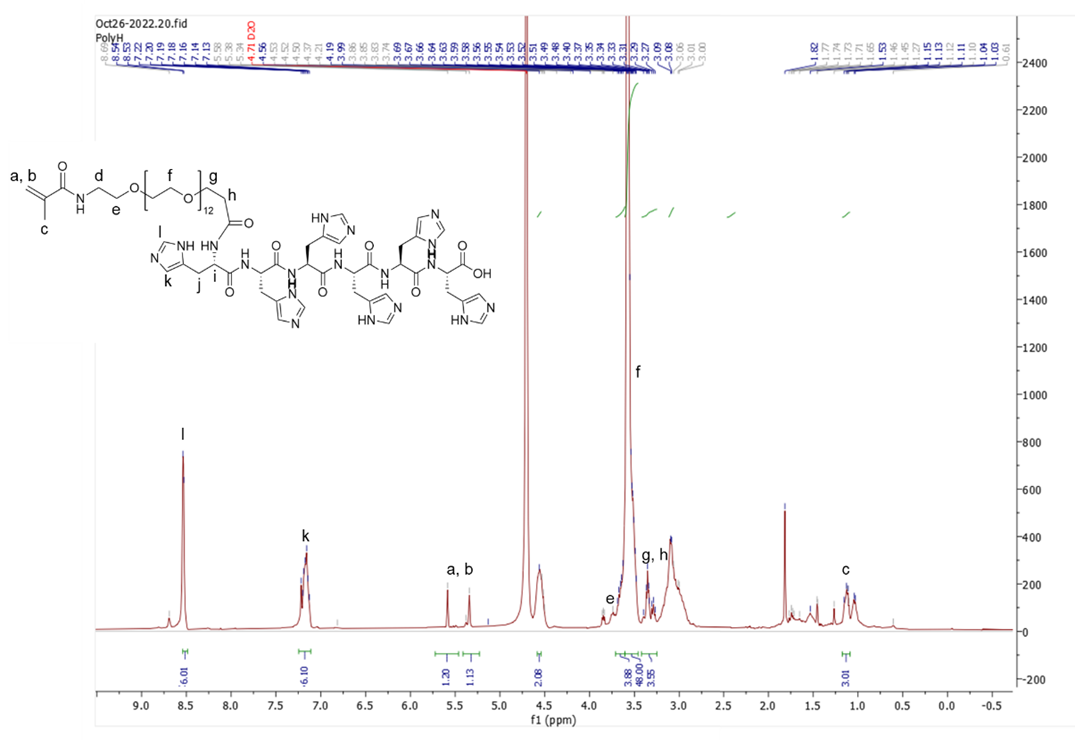
**Figure S7.** ^1^H NMR spectrum of PolyH. ^1^H NMR (400 MHz, D_2_O): δ8.54 (s), δ7.20 (m), δ5.58 (d), δ5.34 (d), δ3.69 (t), δ3.58 (tt), δ3.48 (t), δ3.09 (t), δ1.15 (t).


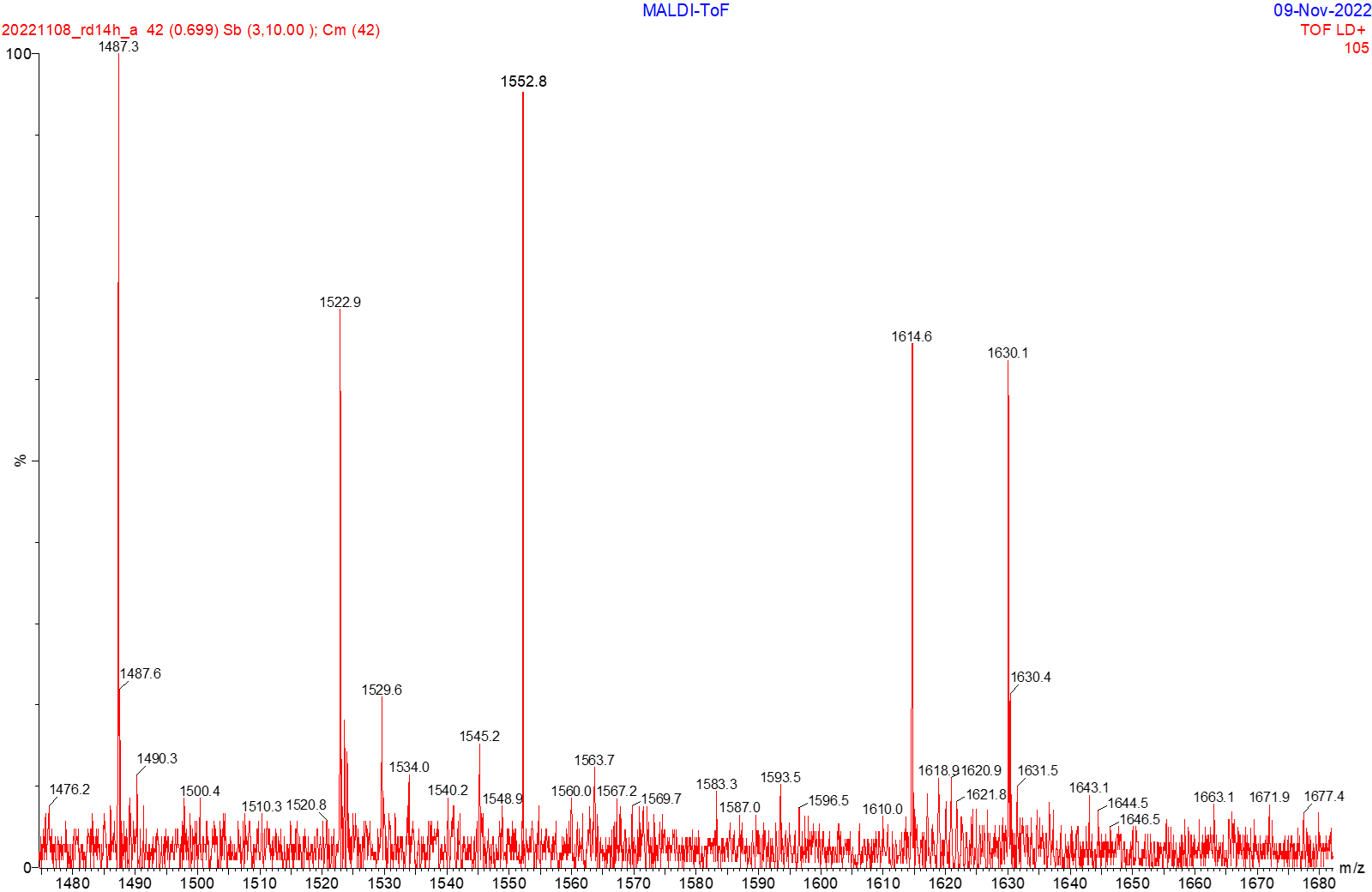
**Figure S8.** Matrix assisted laser desorption ionization-time of flight – mass spectra (MALDI-ToF MS) of acrylated guanidine. M = (C_69_H_105_N_19_O_22_) [M + H]^+^ = 1552.8 m/z.


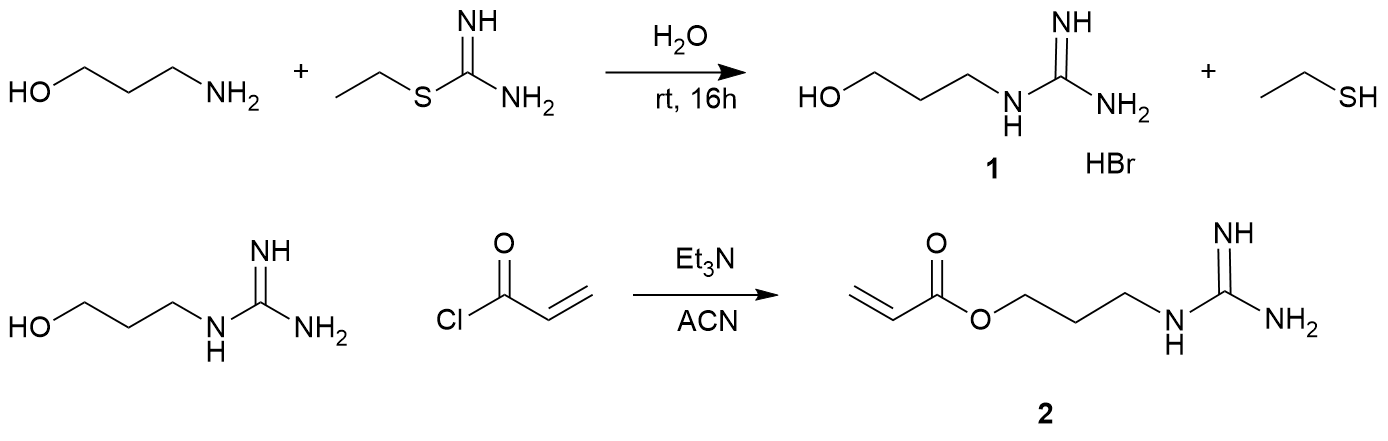


**Figure S9**. Schematic presentation of acrylated-guanidine synthesis.


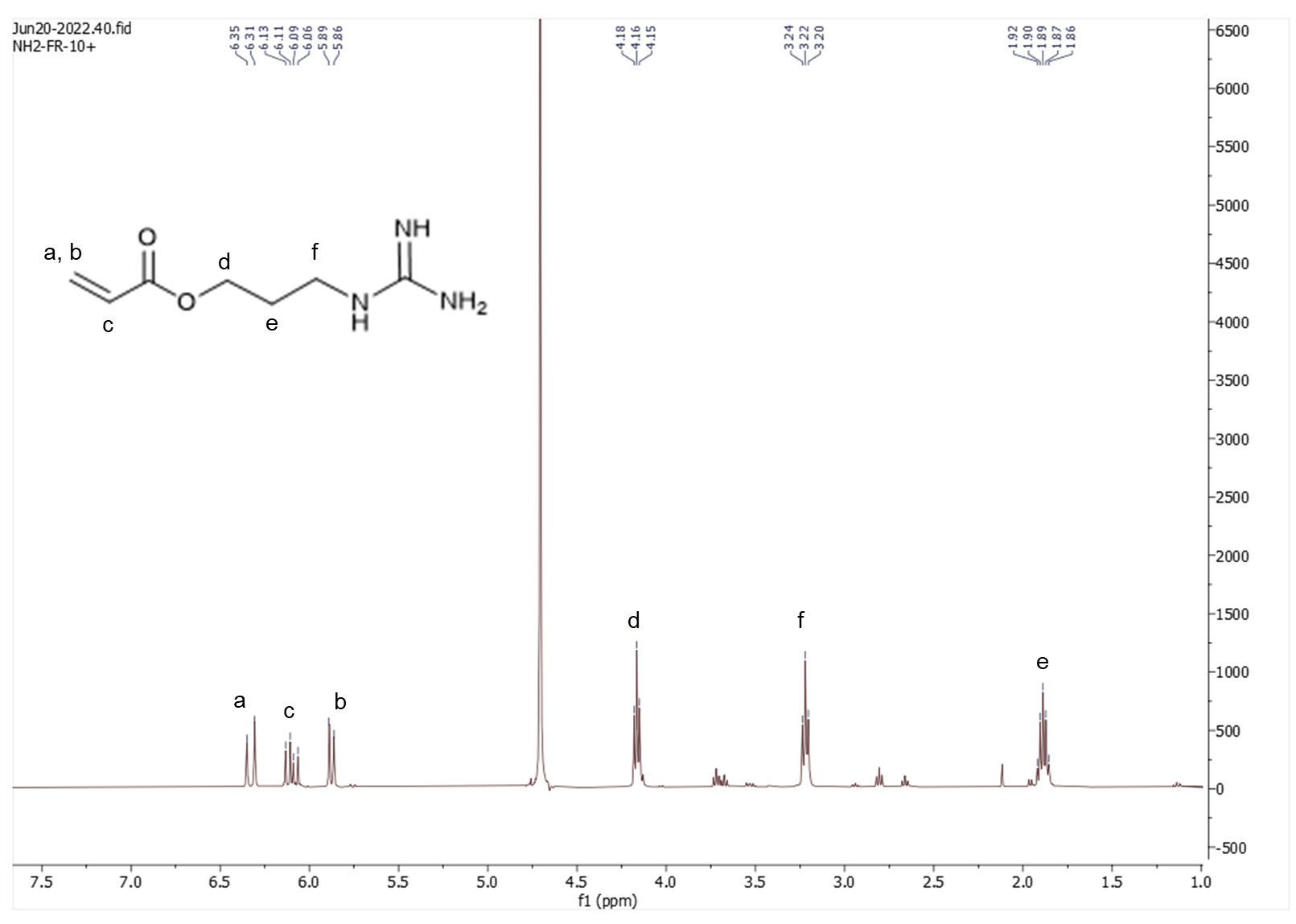
**Figure S10**. ^1^H NMR spectrum of acrylated guanidine (AC-GUA). ^1^H NMR (400 MHz, D_2_O): δ6.35 (d), δ6.11 (dd), δ5.86 (d), δ4.18 (t), δ3.22 (t), δ2.25 (s), δ1.89 (q).


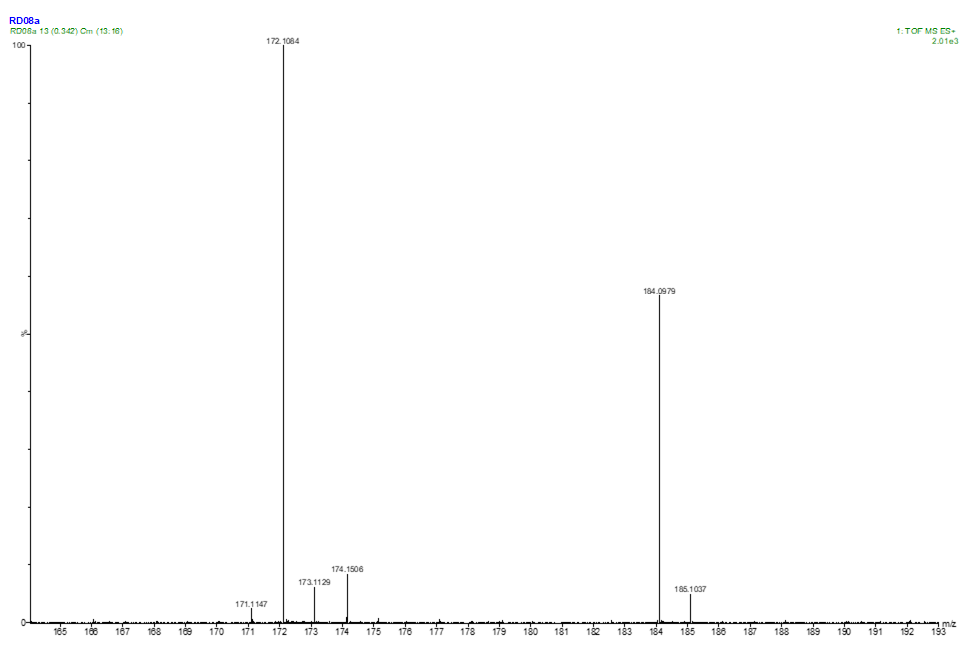
**Figure S11.** Atmospheric pressure chemical ionization – mass spectra of acrylated guanidine. M = (C_7_H_13_N_3_O_2_) [M + H]^+^ = 172.1064 m/z.


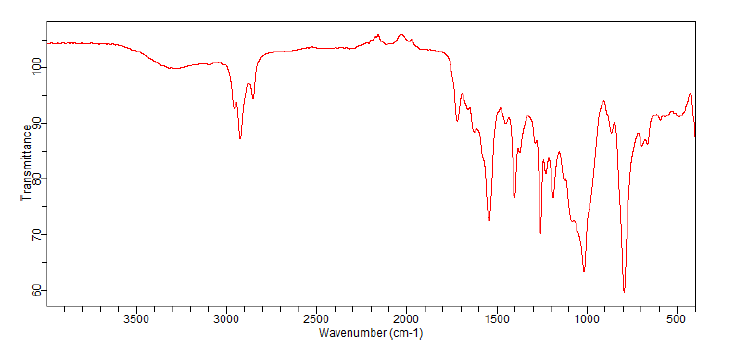
**Figure S12.** Fourier transform infrared spectroscopy (FT-IR) of arcylated guanidine (AC-GUA) N-H stretch: 3700–3500 cm^-1^; alkyl sp^3^ C-H stretch: 2950–2850 cm^-1^ (strong), carbonyl C=O stretch: 1655 cm^-1^ (strong); C-O-C stretch: 1095 cm^-1^ (sharp).


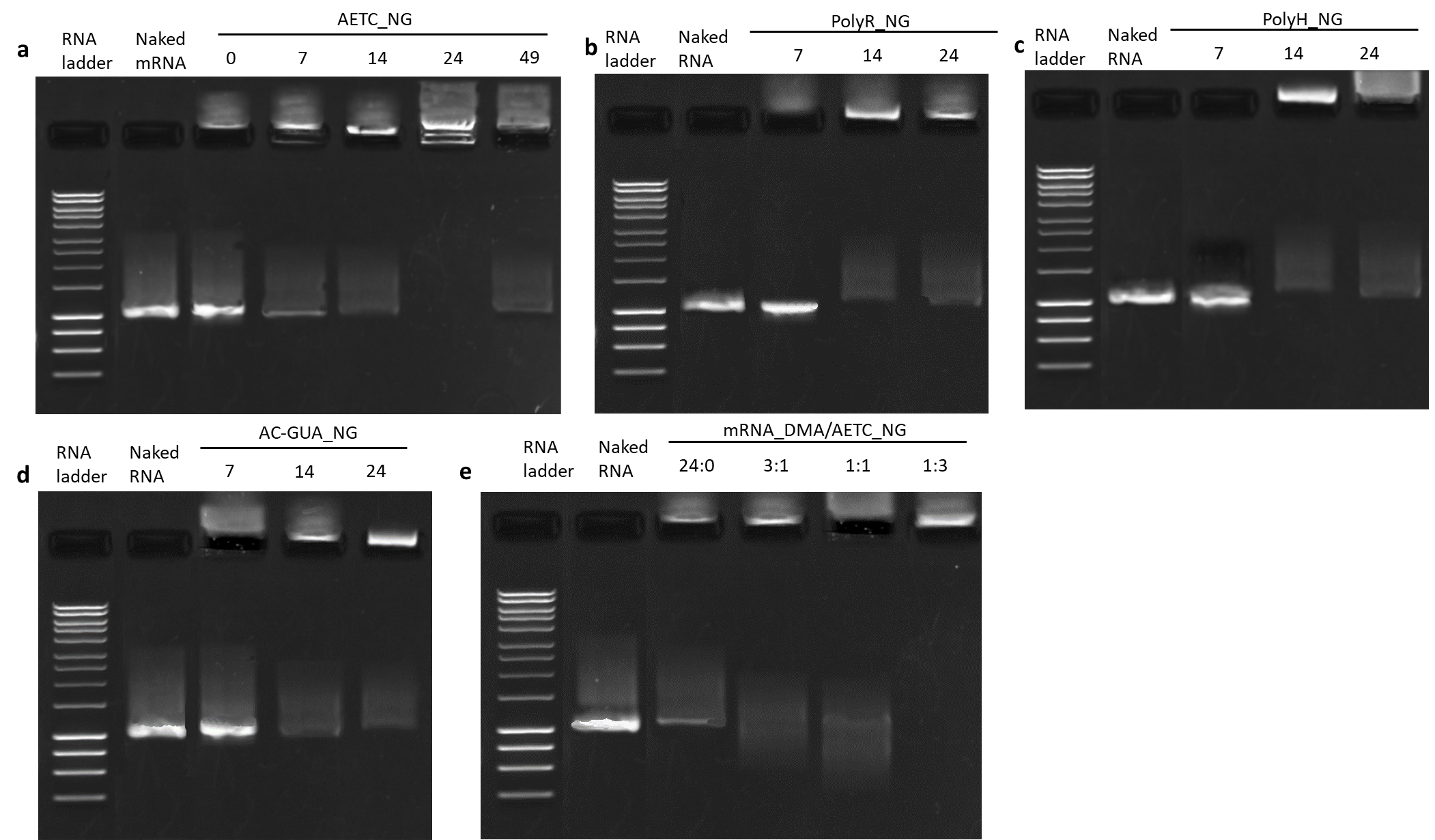
**Figure S13.** Determination of free RNA by the agarose gel electrophoresis assay for a) AETC_NG, b) PolyR_NG, c) PolyH_NG, d) AC-GUA_NG and e) DMA/AETC_NG. In all cases the mol % of RNA complexing monomer added to the NG is indicated by numbers given at the top of the respective lanes, alongside the RNA ladder and unencapsulated, or naked, RNA.


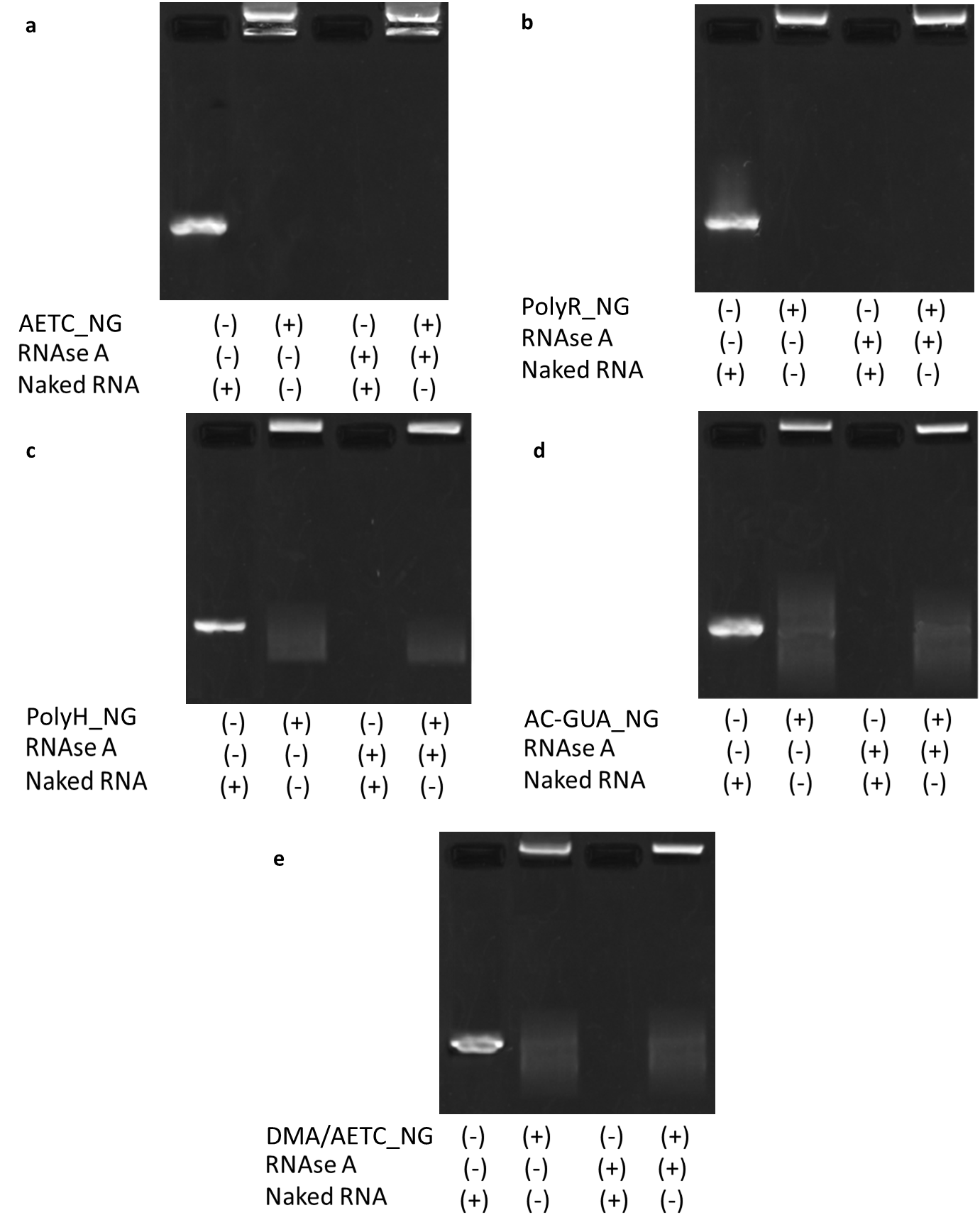


**Figure S14.** Determination of stability of the mRNA entrapped within a) AETC_NG, b) PolyR_NG, c) PolyH_NG, d) AC-GUA_NG and e) DMA/AETC_NG. In all cases, the NGs were treated with RNAse A (100 U/mg) for 2 h at 37 °C.


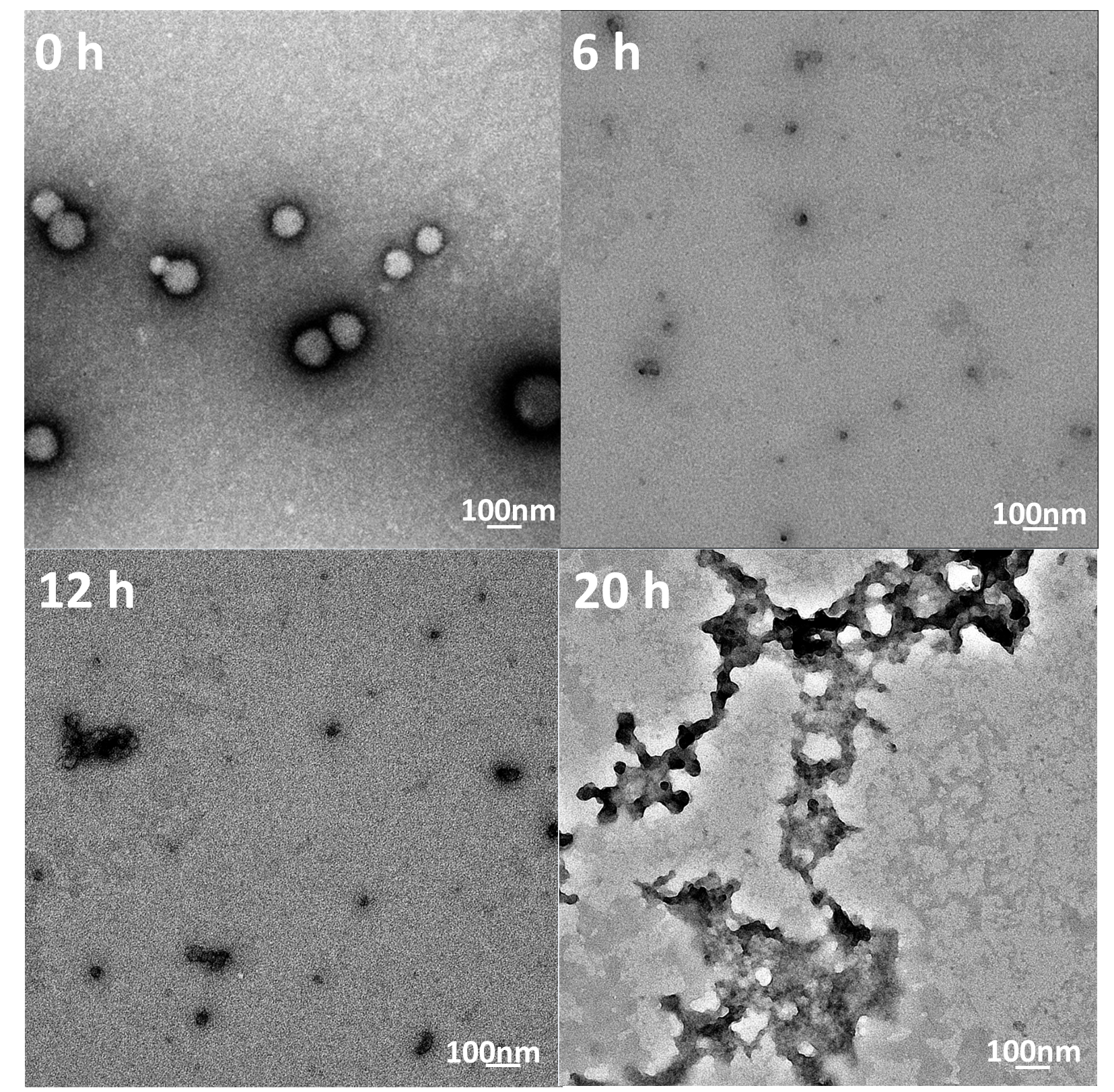


**Figure S15.** Negative stain TEM characterisation of MII-RNA-SS-NG morphological changes upon incubation with 10 mM GSH at various time points (45,000x). Scale bar at 100 nm.


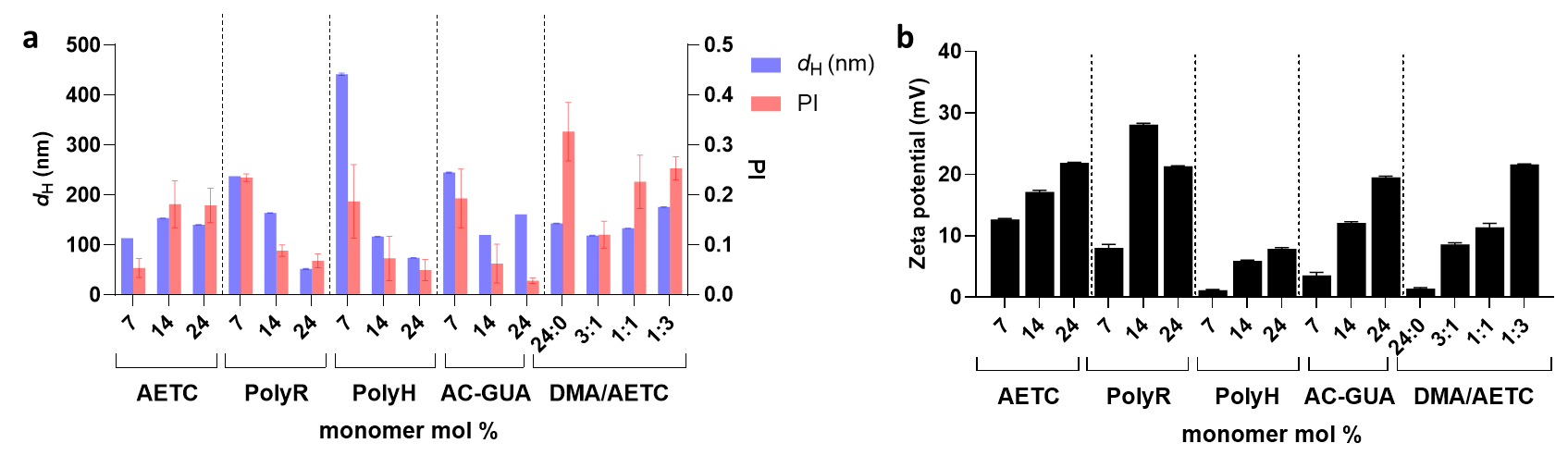
**Figure S16.** Characterization of empty polymeric NGs. a) Physicochemical characterization of size (hydrodynamic diameter) and polydispersity (PI) as obtained from dynamic light scattering measurements. For each figure the ratios displayed on the x axes relate to the mol % of the respective monomers incorporated within the NGs. (b) Zeta potential (mV) of synthesized NGs as obtained from electrophoretic scattering measurements (measured in water, pH 7.2).


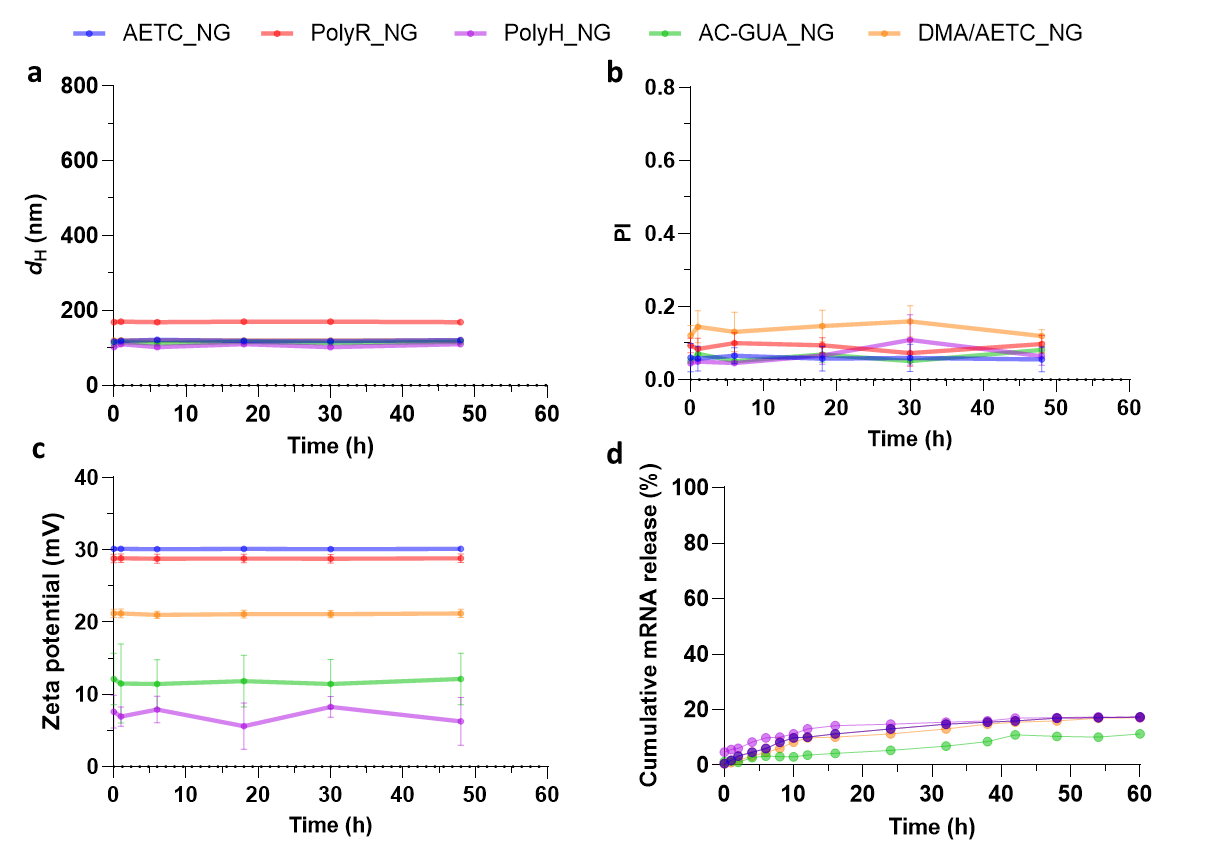


**Figure S17.** Characterization of NG structural changes in the absence of GSH. Physicochemical characterization of NG a) size (hydrodynamic diameter), b) polydispersity (PI) and c) zeta potential measurements of NGs in the absence of GSH. d) Cumulative RNA release from NGs incubated at 37 °C in the absence of GSH. In all cases, data are presented as mean ± standard deviation for n = 3.


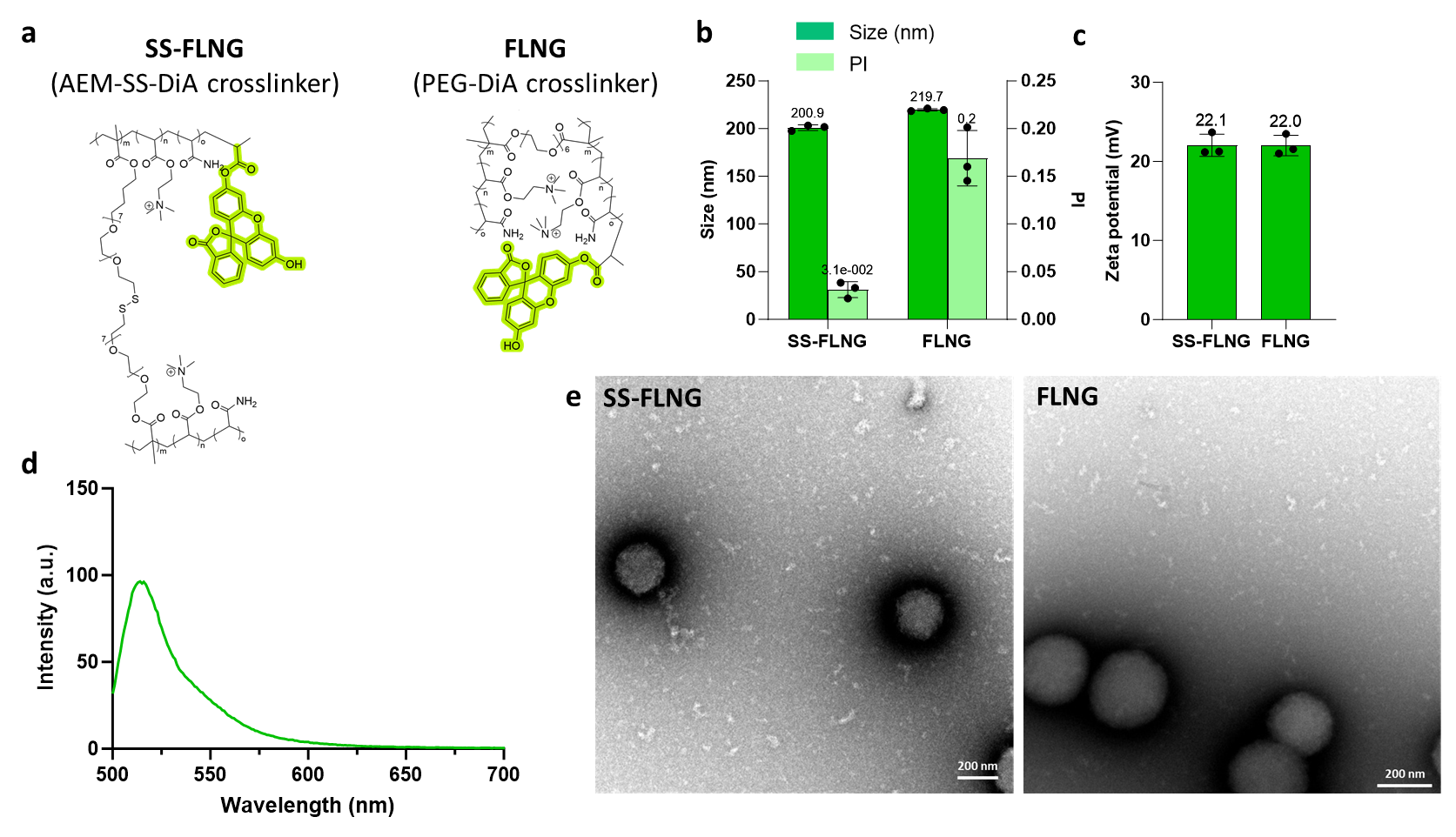
Figure S18. Characterisation of disulphide (SS-FLNG) and non-disulphide (non-SS-FLNG) fluorescent nanogels (n = 3). a) Proposed chemical structure of the nanogels synthesised with the two different crosslinkers with the disulphide crosslinker containing-nanogel. The fluorescent monomer is shown in green. b) Graph comparing size and polydispersity of synthesised nanogels. c) Zeta potential of synthesised nanogels as obtained from DLS analysis. d) Emission spectra for 100 µg/mL fluorescent nanogel in water, pH 7.2. e) Negative stain TEM of SS-FLNG and FLNG (45,000 x).

**
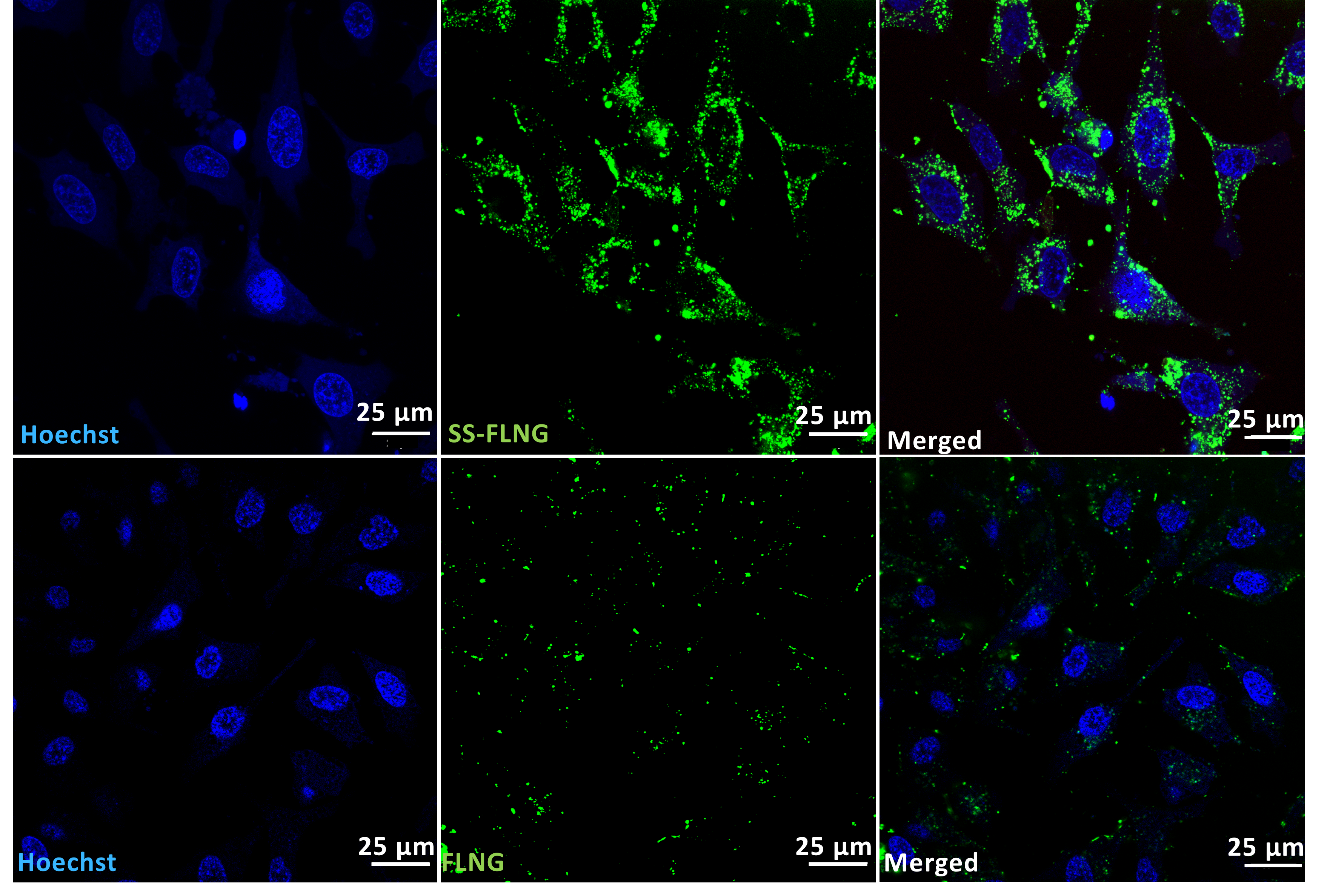
Figure S19.** Confocal microscopy images showing the intracellular uptake of fluorescein-labelled disulphide (SS-FLNG) and non-disulphide NGs (FLNG) in HeLa cells fixed 8 h post-treatment, stained with 1 µg/mL Hoechst 33342. All images were taken with 63 x oil immersion objective. Scale bar at 25 μm.

**
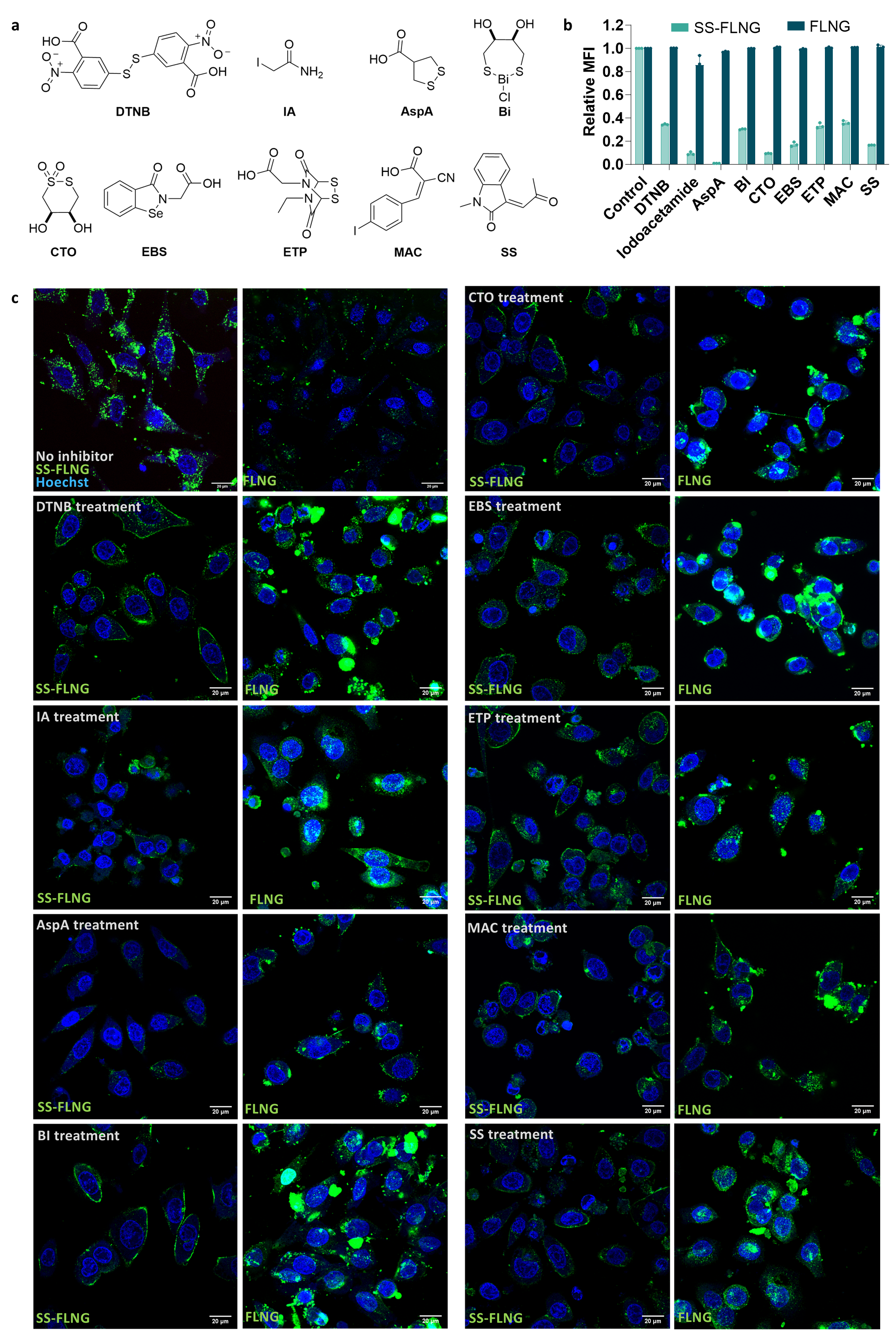
**Figure S20. Screening of thiol-mediated uptake inhibitors. a) Chemical structures of inhibitors used in the study, including DTNB (5,5-dithio-bis-(2-nitrobenzoic acid)), IA (iodoacetamide), AspA (asparagusic acid), BI ((5*S,*6*R*)-2-chloro-1,3,2-dithiabismepane-5,6-diol), CTO (cyclic thiosulfonate), EBS (ebselen analog), ETP (epidithiodiketopiperazine), MAC (michael acceptor) and SS (super-spice). b) Effect of various inhibitors on nanogel internalization, as assessed by flow cytometry (n = 3). c) Confocal microscopy images showing the intracellular uptake of fluorescein-labelled disulphide (SS-FLNG) and non-disulphide nanogels (FLNG) in HeLa cells treated with different inhibitors, stained with 1 µg/mL Hoechst 33342. All images were taken with 63 x oil immersion objective. Scale bar at 25 μm. Data shown are mean ± SD.

**
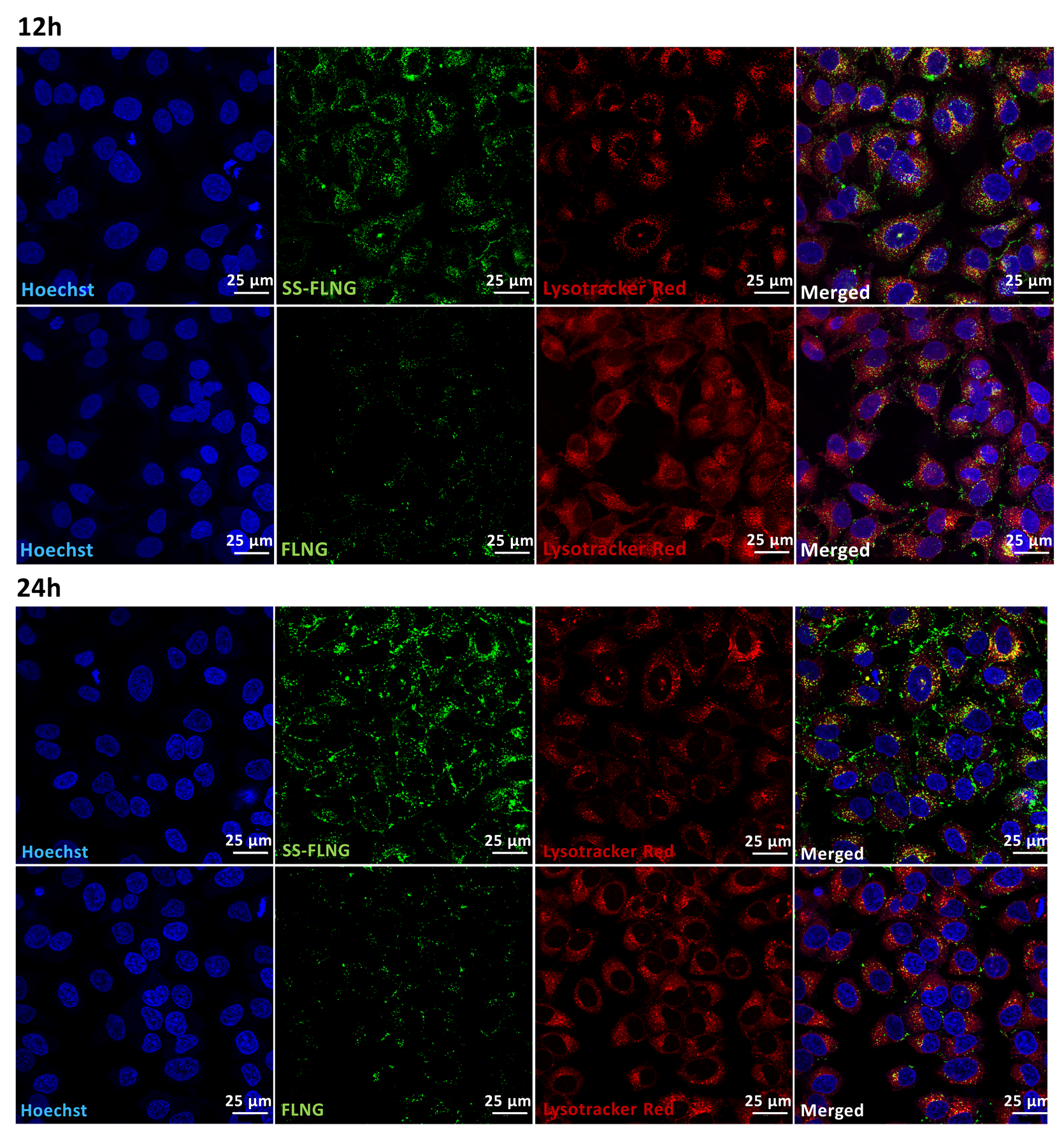
Figure S21.** Evaluation of endosomal escape in HeLa cells. Confocal microscopy images showing the intracellular uptake of disulphide (SS-FLNG) and non-disulphide (FLNG) fluorescent NGs at 12 h and 24 h. Cell stained with 75 nM Lysotracker Red and 1 µg/mL Hoechst 33342. All images were taken with 63 x oil immersion objective. Scale bar at 25 μm.

**
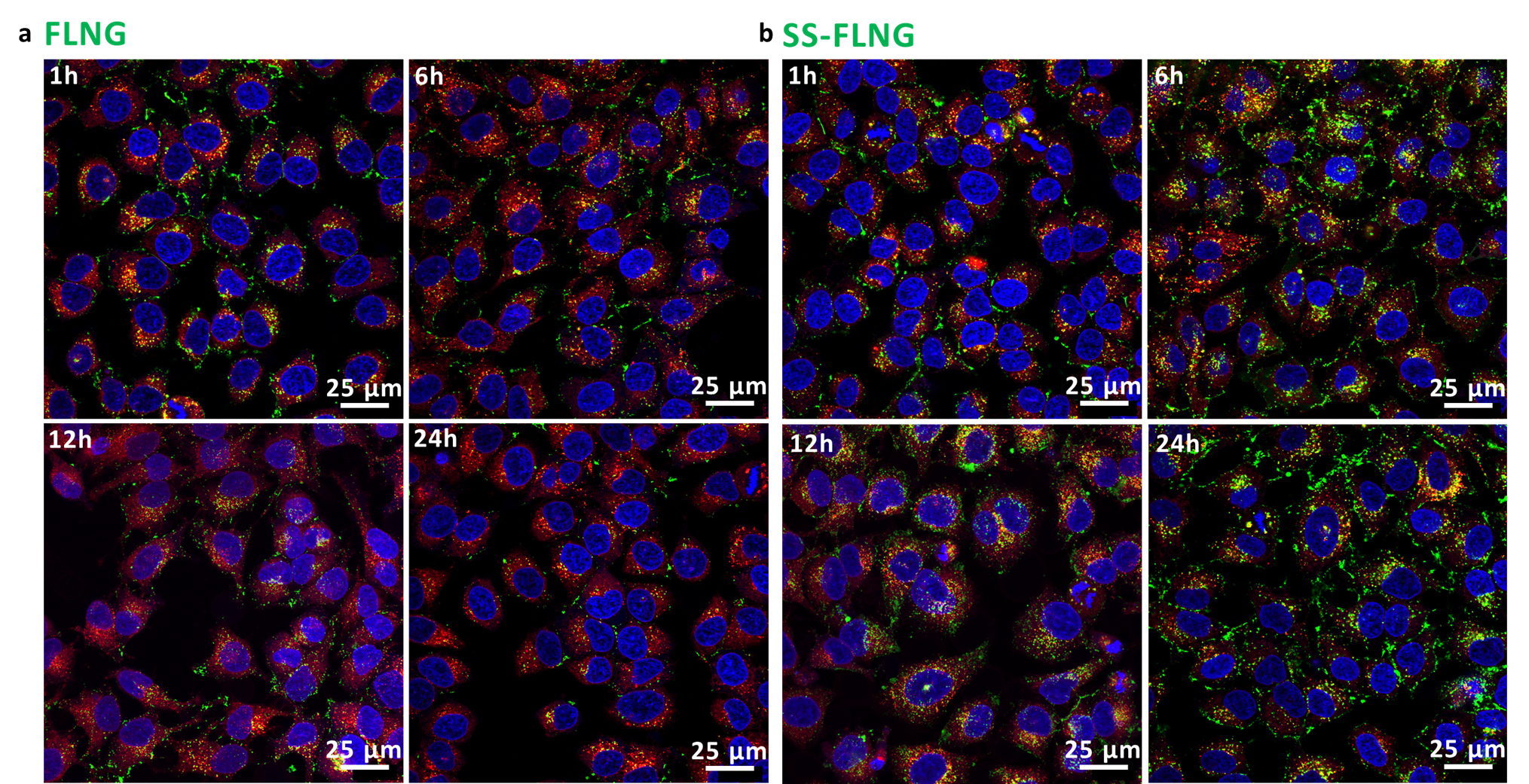
**

**Figure S22.** a) Time dependent monitoring of endosomal escape of FLNGs using confocal microscopy. b) Time dependent monitoring of endosomal escape of SS-FLNGs using confocal microscopy. Lower colocalization of FLNG and Lysotracker Red were observed with time which indicate endosomal escape.

**
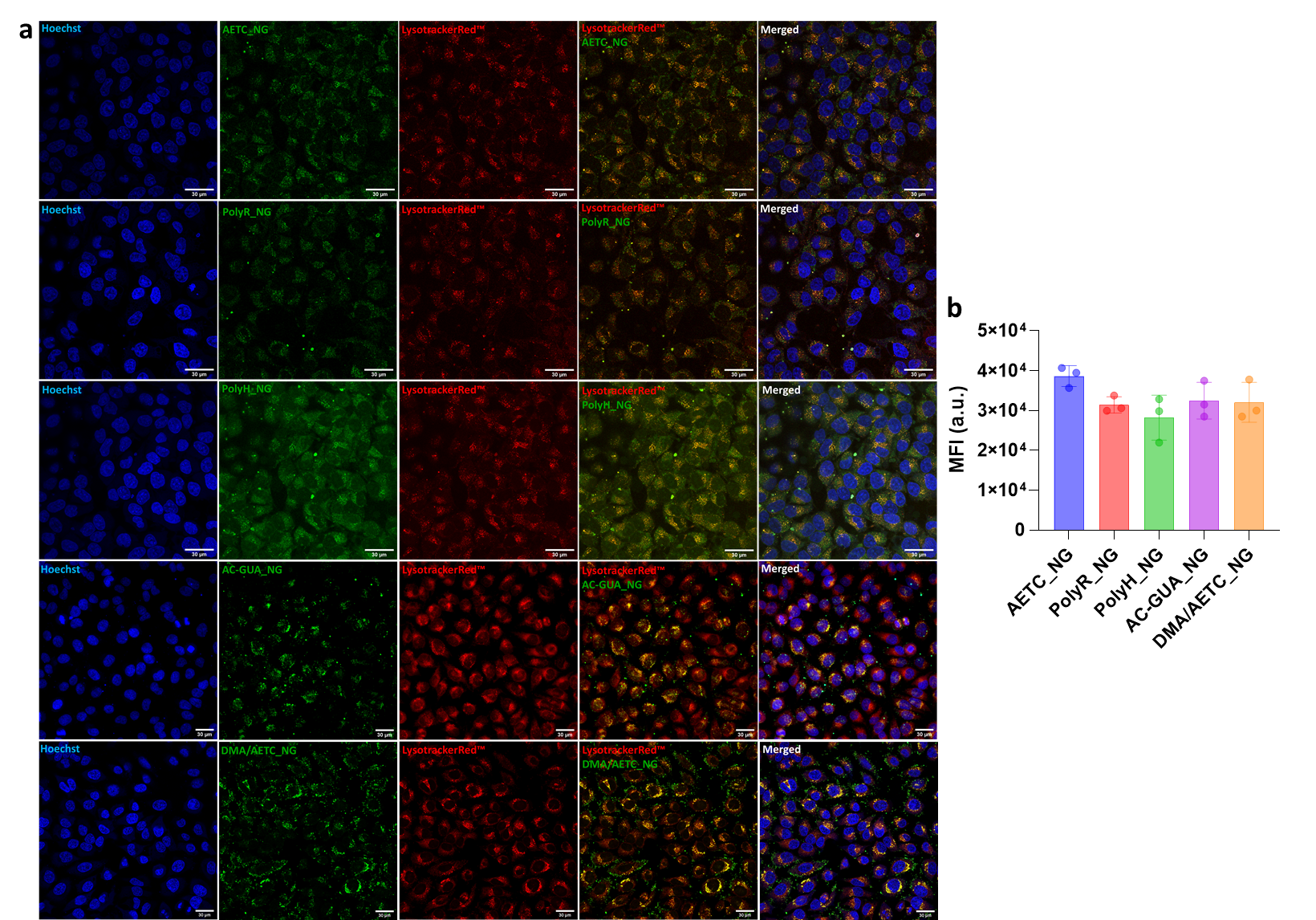
**

**Figure S23.** a) Maximum projection confocal microscopy images showing the intracellular uptake of fluorescently labelled (green) AETC_NG, PolyR_NG, PolyH_NG, AC-GUA_NG and DMA/AETC_NG. Cells were stained 75 nM LysotrackerRed™ (red) and 1 µg/mL Hoechst 33342 (blue). All images were taken with 40 x oil immersion objective. Scale bar at 30 μm. b) Quantification of intracellular uptake of the different NGs by microscopy. Mean fluorescence intensities (MFI %) are shown (n = 3). Data shown are mean ± SD.


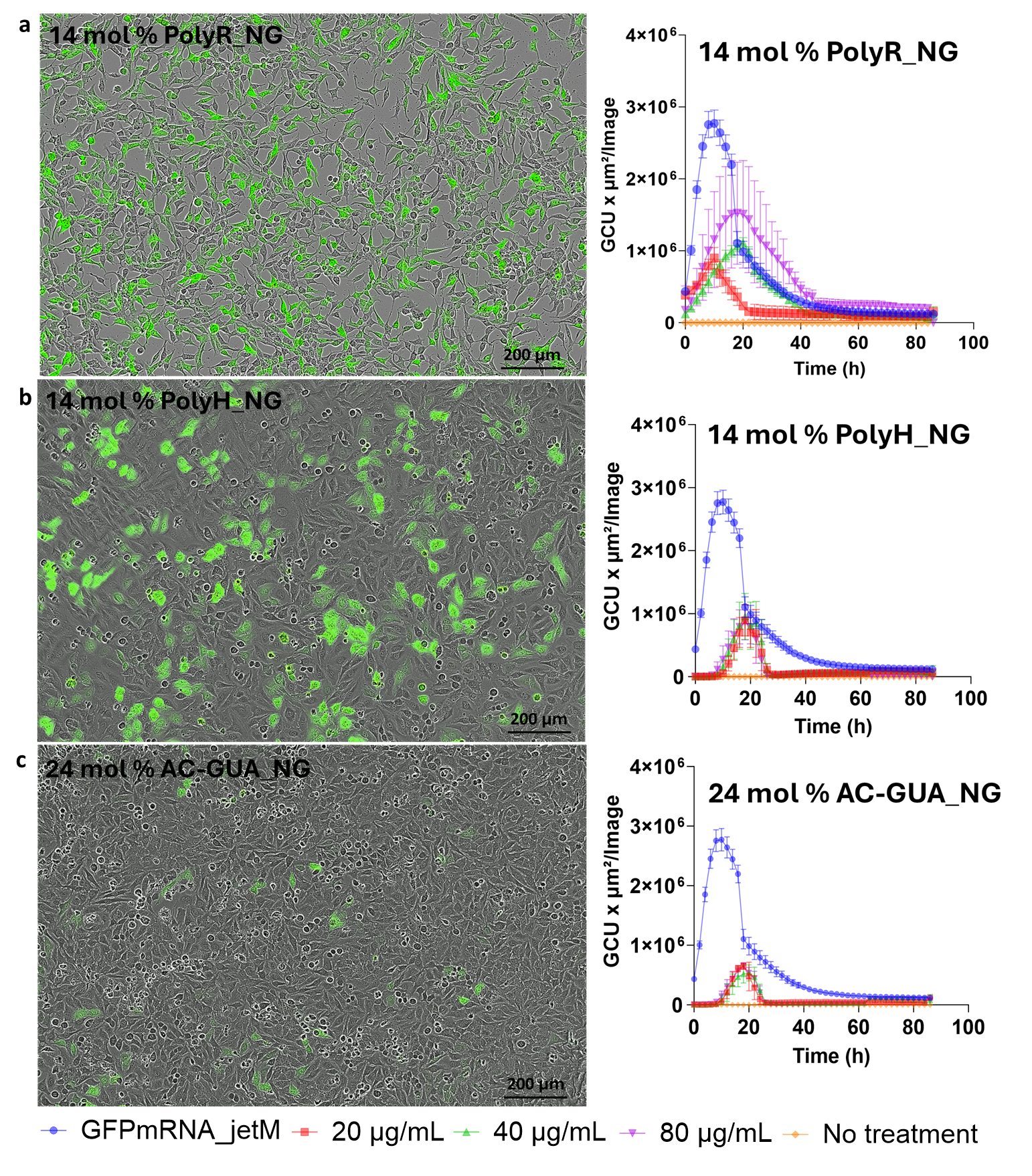


**Figure S24.** *In vitro* NG GFP mRNA expression profiles with associated IncuCyte images of GFP expression in HeLa cells treated with 80 µg/mL of NGs at 20 h for a) PolyR_NG, b) PolyH_NG and c) AC-GUA_NG. Each expression profile shows the fluorescence expression (green calibrated unit x µm^2^/image) as a function of time. In all cases, transfection efficiency was compared with the commercial reagent Polyplus® jetMESSENGER®. Scale bar for the images at 200 µm.

**Figure S25.** Cell viability determined for all NGs by varying concentrations in HeLa cells. In all cases, data are presented as mean ± standard deviation for n = 3.


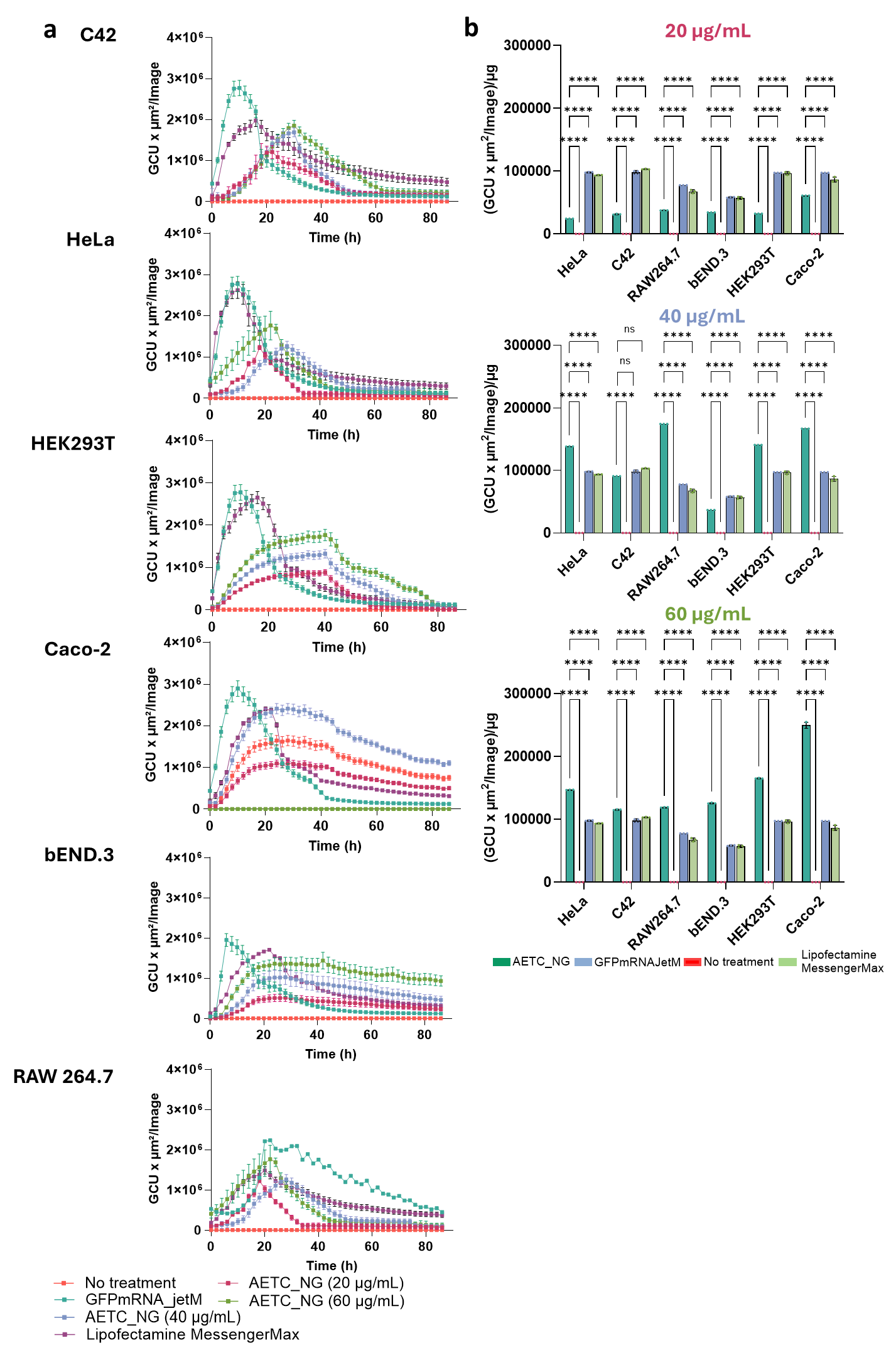
**Figure S26.** Comparing transfection efficiency of varying doses GFPmRNA_AETC_NG with Polyplus® jetMESSENGER® and LipoMessenger Max. a) The graphs show the fluorescence expression (green calibrated unit x µm^2^/image) as a function of time for each cell line for treated and untreated cells. b) Fluorescent protein expression normalized by total protein quantification (BCA assay, details in Methods section) for each mRNA_AETC_NG dose, compared against untreated cells and cells treated with the commercial reagent Polyplus® jetMESSENGER®. Data analysed and evaluated for statistical significance by employing a a two-way ANOVA with Tukey’s multiple comparisons test (ns < 0.1234, *p < 0.0332, **p < 0.0021, ***p < 0.0002, ****p < 0.0001).


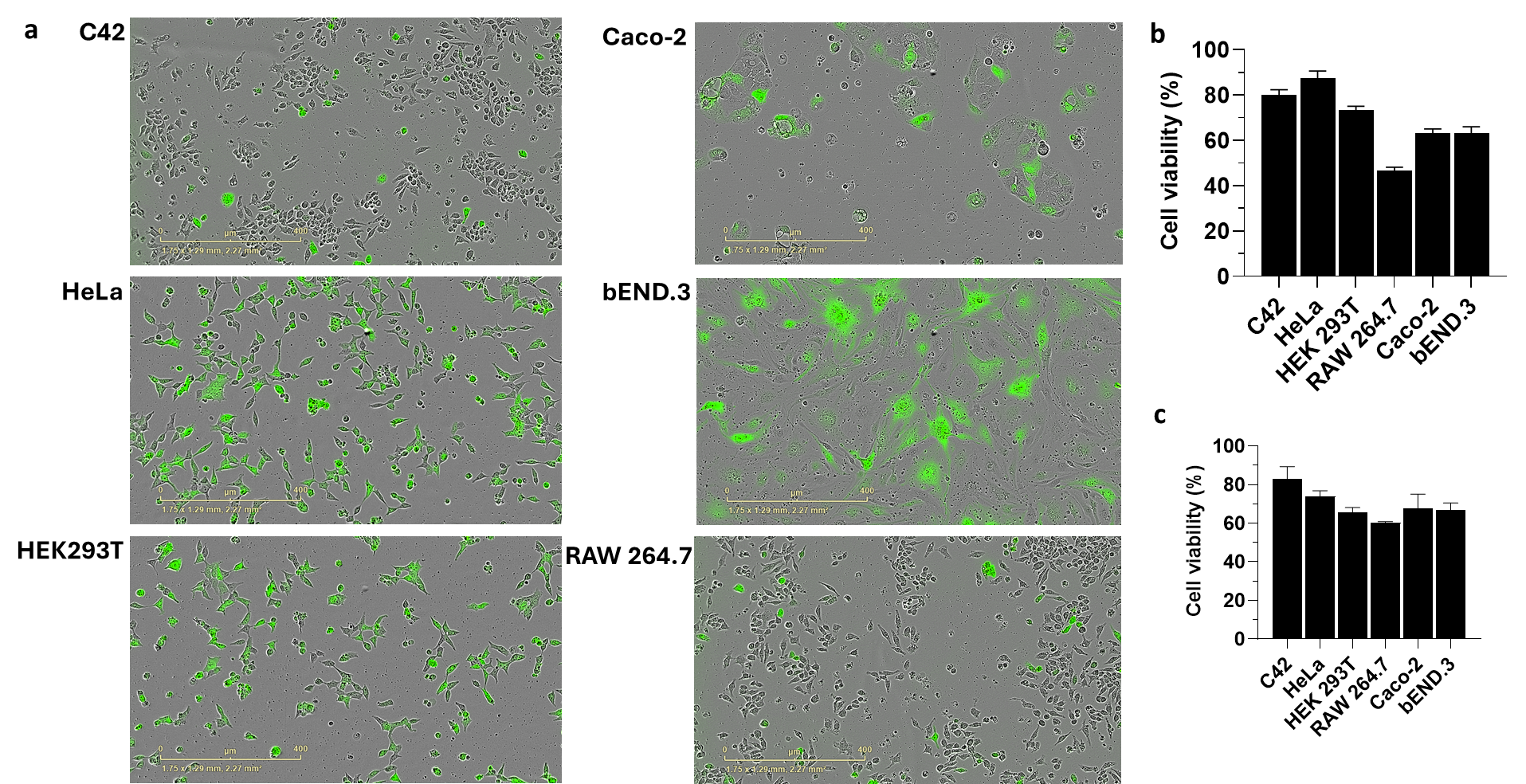


**Figure S27.** a) Representative IncuCyte images of each cell line transfected with Polyplus® jetMESSENGER®, at the 20 h time point. Scale bar is at 44 µm. Cell viability determined for b) PolyPlus® Jetmessenger® (0.25 µL) and c) LipoMessenger Max (1 µL) in different cell lines. In all cases, data are presented as mean ± standard deviation for n = 3.

**Figure S28.** Haemolysis percentages of AETC_NGs at different concentrations between 5 – 500 µg/mL, incubated with RBCs for various timepoints. In all cases, data are presented as mean ± standard deviation for n = 3.


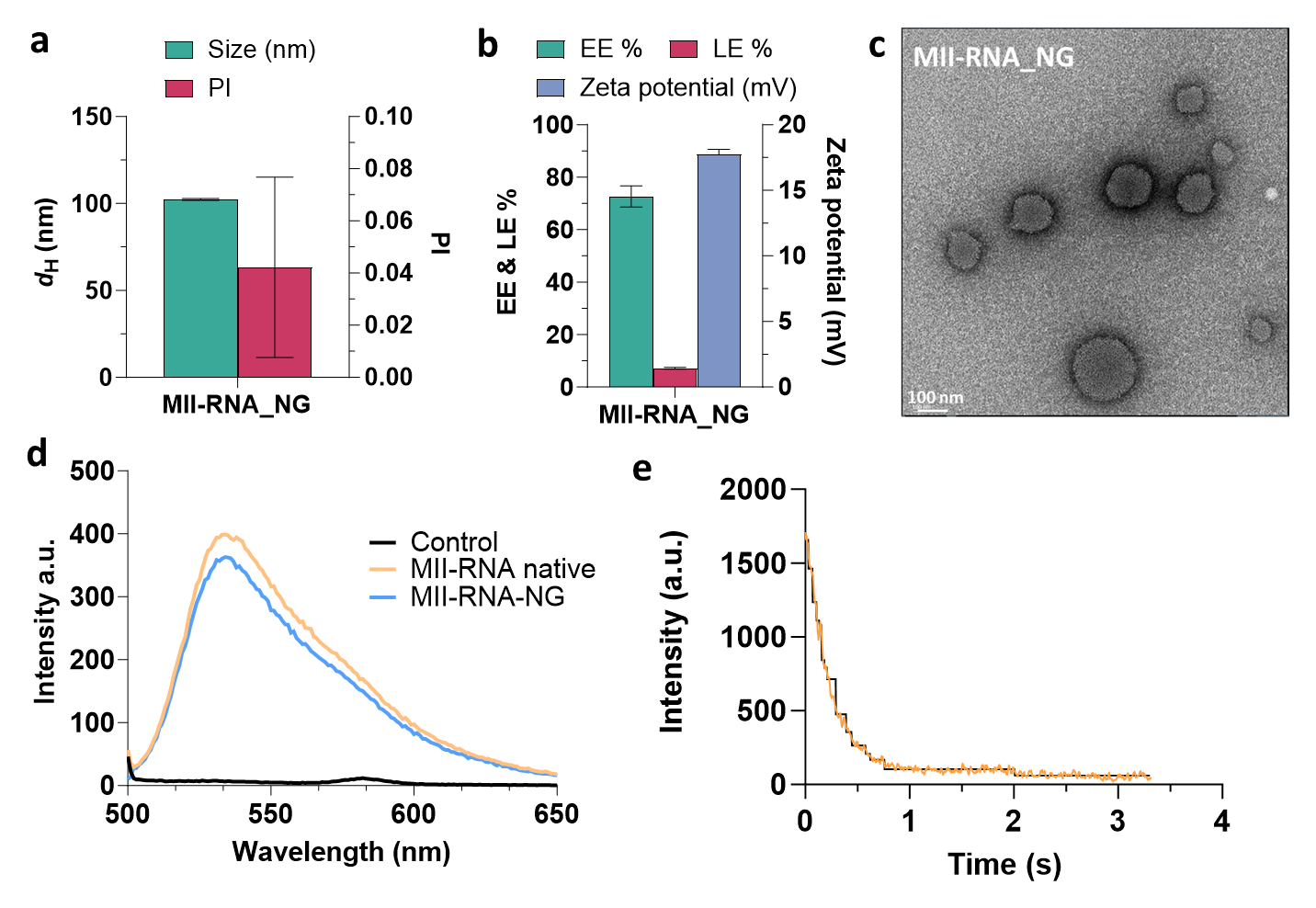


**Figure S29.** Comparison of NG structural characteristics against commercial reagents. a) Negative stain TEM of MII-RNA-loaded NGs, and PolyPlus jetMESSENGER® reagent (45,000 x). Scale bar at 100 nm. b) Graph comparing NG size and polydispersity against commercial transfection reagents. c) Graph comparing encapsulation and loading efficiencies and zeta potential of NGs against commercial transfection reagents. d) Emission spectra for 125 nM MII-RNA encapsulated within NGs and unencapsulated native RNA, in the presence of 200 nM TO1-Biotin. PKM buffer is shown as a control. e) Representative bleaching curve for an individual MII x9-RNA foci (yellow) fitted with a maximum likelihood step fitting algorithm (black).


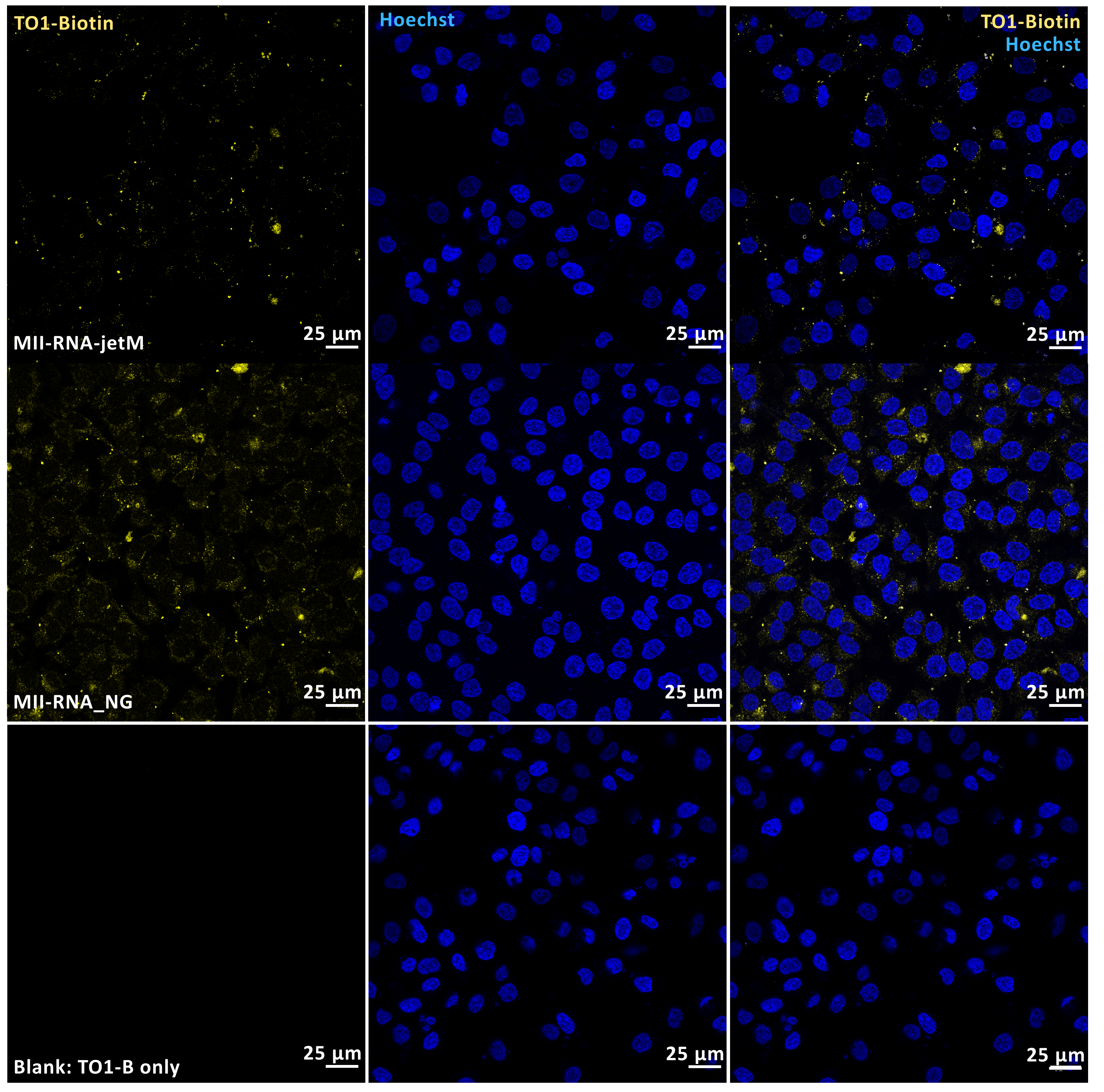
Figure S30. Maximum projection of HeLa cells treated with MII-RNA-jetM, MII-RNA_NG and fixed at 12 h post-transfection, stained with 200 nM TO1-B (yellow) and 1 µg/mL Hoechst 33342. Control cells treated with only 200 nM TO1-B and no Mango II RNA also shown. All images were taken with 40 x oil immersion objective.


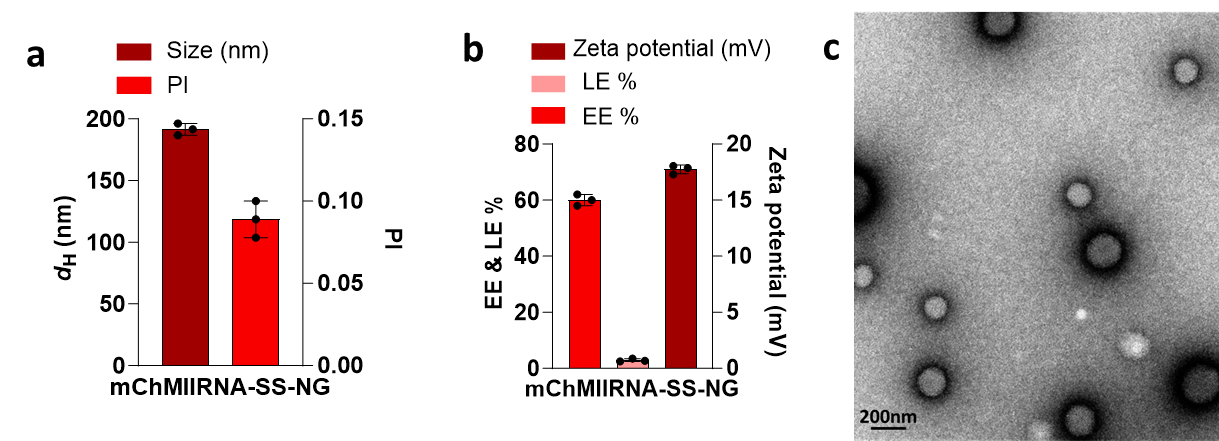
**Figure S31.** Characterisation of mChMII-RNA-loaded NGs and transfection. a) Size and size distribution of mChMII-RNA-SS-NG. b) Zeta potential, encapsulation and loading efficiencies of mChMII-RNA-SS-NG. c) Negative stain TEM of mChMII-RNA-SS-NG (45,000 x).


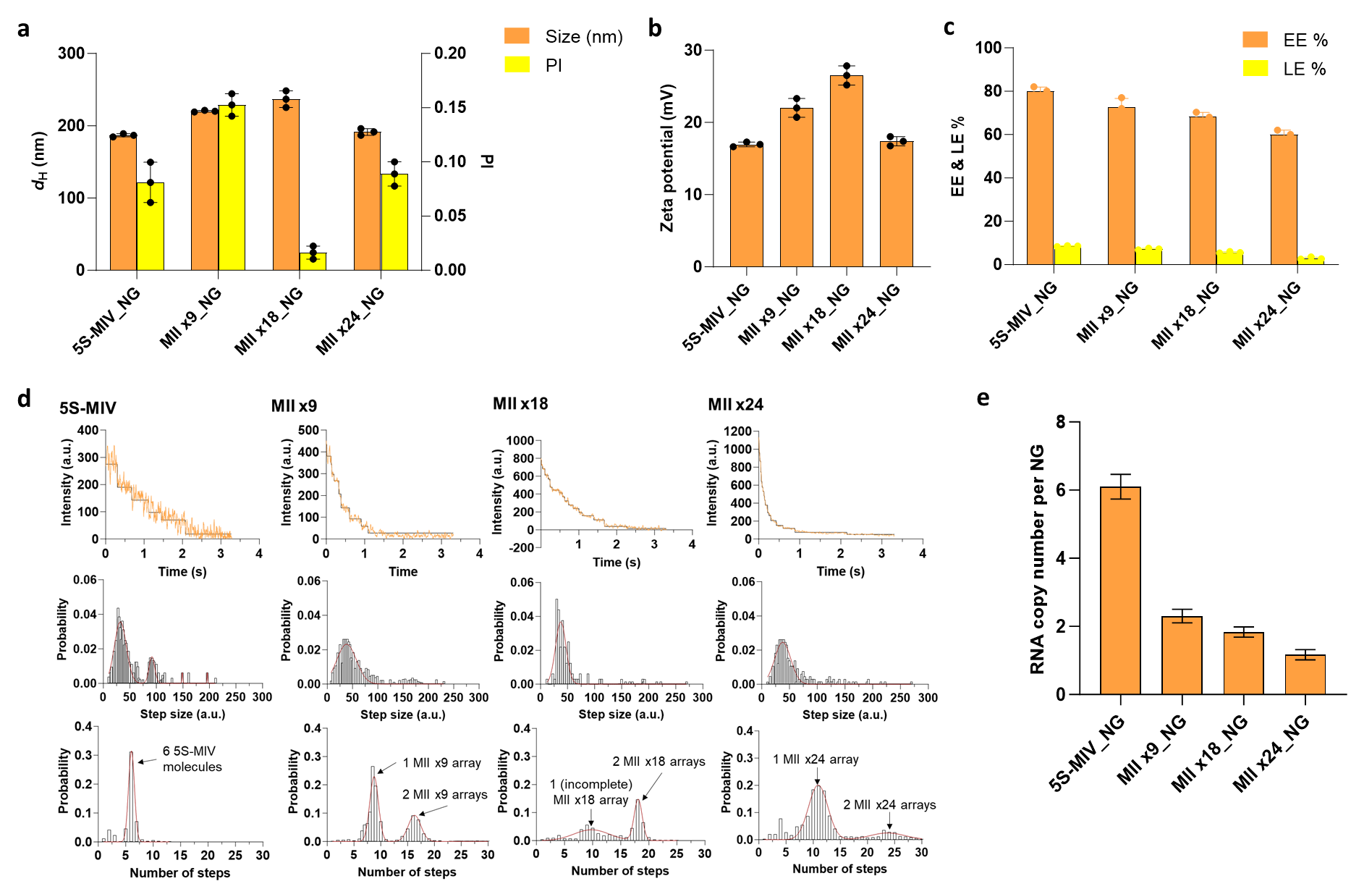


Figure S32. Graphs comparing a) sizes and polydispersities of nanogels, b) zeta potentials, c) encapsulation and loading efficiencies of nanogels loaded with various Mango RNA payloads. d) Representative bleaching curves for individual Mango RNA foci (yellow) fitted with a maximum likelihood step finding algorithm (black) with associated distributions of photobleaching step sizes and number of steps fitted with gaussian curves. Note: the incomplete MII x18 array could have resulted from incomplete transcription of the aptamer repeat sequence, which can be difficult to transcribe via *in vitro* methods. e) RNA copy number per NG as estimated from the step-wise photobleaching analysis.

**
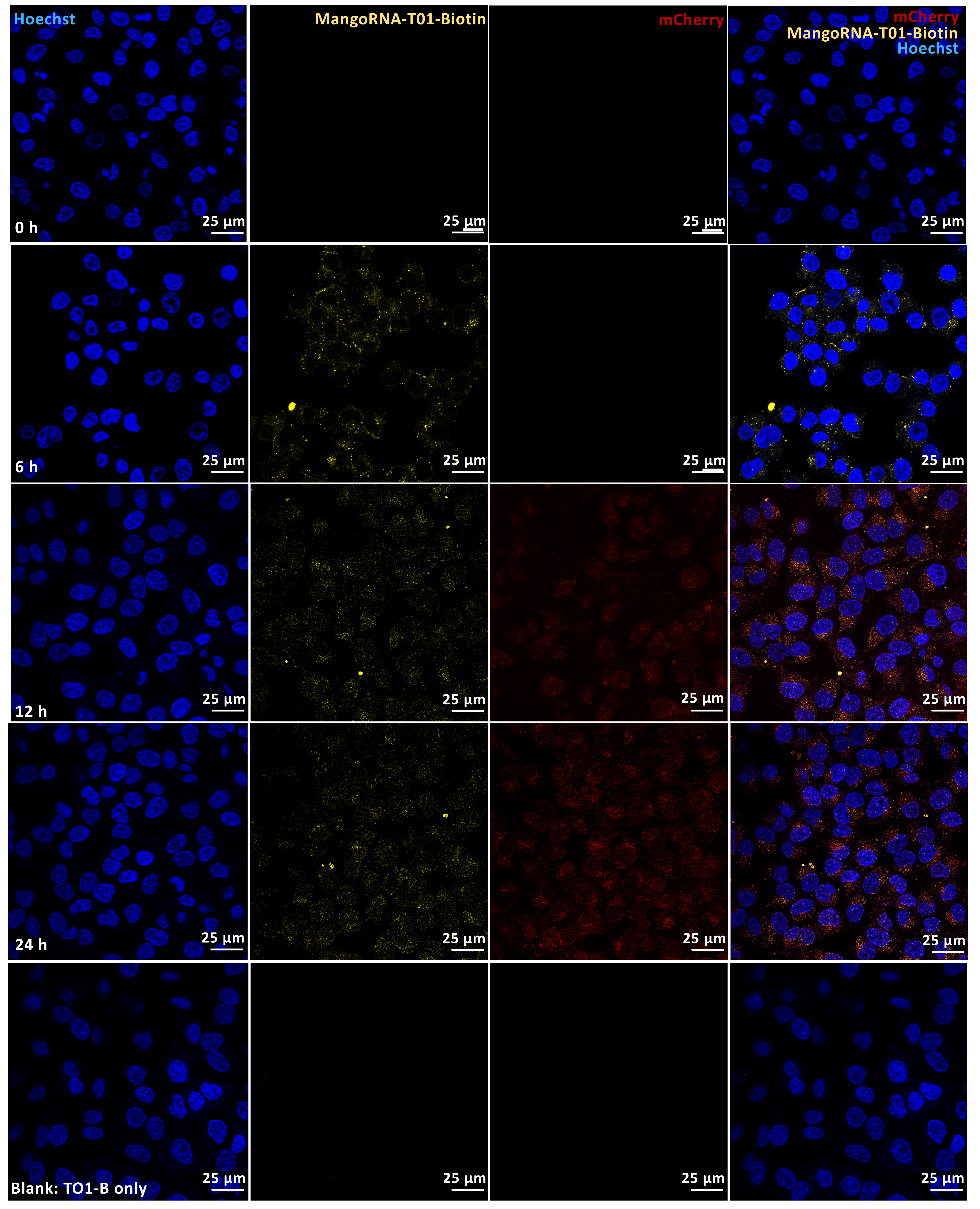
**Figure S33. Maximum projection of HeLa cells fixed at various timepoints post-transfection with the mChMII-RNA-SS-NG, where the mCherry expression is shown in red, stained with 200 nM TO1-B (yellow) and 1 µg/mL Hoechst 33342. Control cells treated with only 200 nM TO1-B and no MII-RNA also shown. All images were taken with 63 x oil immersion objective.


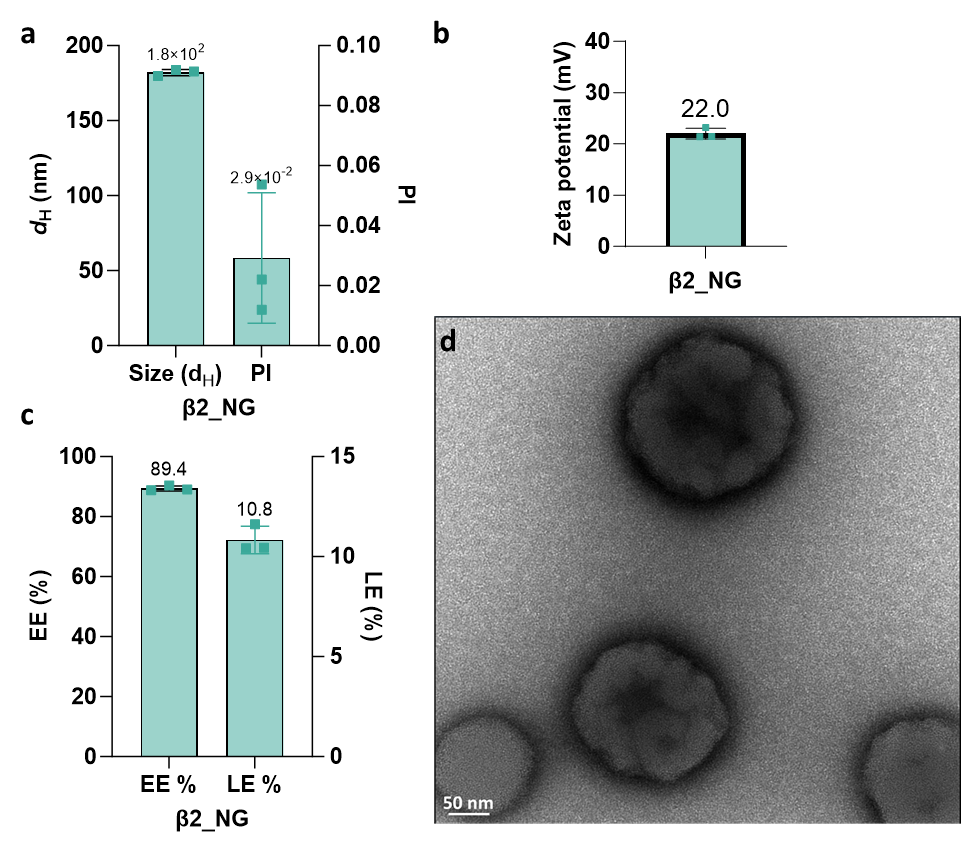
Figure S34. Physicochemical characterization of β2 mRNA-loaded AETC nanogels (β2_NG). a) Size (hydrodynamic diameter) and polydispersity (PI) as obtained from dynamic light scattering measurements. b) Zeta potential (mV) of synthesised NGs as obtained from electrophoretic scattering measurements (measured in water, pH 7.2). c) Encapsulation and loading efficiency of β2 mRNA within the NGs. d) Negative stain transmission electron micrographs of β2_NGs (75,000 x). Scale bar at 50 nm. In all cases, data are presented as mean ± standard deviation for n = 3.

|  | **Nanogel** | **Feed composition** | | | | **Initiating system** | **Nanogel structural features** | | |
| --- | --- | --- | --- | --- | --- | --- | --- | --- | --- |
|  |  | **AM** | **Cationic monomer** | | **SS-DiA** | **APS/TEMED** | **Size (nm)** | **PI** | **Zeta potential (mV; pH 7.4, water)** |
| **Cationic monomer type** | **no.** | **mol%** | | | | **mol%** |  | | |
| **AETC** | **1** | **90.7** | **0** | | **7.0** | **15** | **126.4 ± 4.0** | **0.033 ± 0.009** | **-8.5 ± 1.5** |
|  | **2** | **83.56** | **7.14** | | **7.0** | **15** | **198.8 ± 17.7** | **0.010 ± 0.056** | **6.6± 0.3** |
|  | **3** | **77.2** | **13.5** | | **7.0** | **15** | **144.3 ± 1.3** | **0.042 ± 0.020** | **10.5± 1.6** |
|  | **4** | **66.9** | **23.8** | | **7.0** | **15** | **118.1 ± 11.7** | **0.072 ± 0.062** | **22.5± 0.5** |
|  | **5** | **41.5** | **49.2** | | **7.0** | **15** | **247.2 ± 2.8** | **0.336 ± 0.018** | **30.5± 7.0** |
| **PolyR** | **7** | **85.86** | **7.14** | | **7.0** | **15** | **239.4 ± 2.7** | **0.232 ± 0.007** | **8.3 ± 0.6** |
|  | **8** | **79.5** | **13.5** | | **7.0** | **15** | **168.9 ± 2.1** | **0.094 ± 0.020** | **28.8 ± 0.6** |
|  | **9** | **69.2** | **23.8** | | **7.0** | **15** | **54.8 ± 0.9** | **0.283 ± 0.004** | **21.2 ± 0.6** |
| **PolyH** | **10** | **85.86** | **7.14** | | **7.0** | **15** | **488.5 ± 6.1** | **0.162 ± 0.010** | **1.8 ± 0.8** |
|  | **11** | **79.5** | **13.5** | | **7.0** | **15** | **111.8 ± 0.5** | **0.051 ± 0.003** | **5.6 ± 4.2** |
|  | **12** | **69.2** | **23.8** | | **7.0** | **15** | **77.3 ± 21.8** | **0.461 ± 0.059** | **7.8 ± 0.7** |
| **AC-GUA** | **13** | **85.86** | **7.14** | | **7.0** | **15** | **254.3 ± 21.5** | **0.135 ± 0.027** | **3.8 ± 0.8** |
|  | **14** | **79.5** | **13.5** | | **7.0** | **15** | **111.8 ± 0.5** | **0.051 ± 0.003** | **10.8 ± 3.9** |
|  | **15** | **69.2** | **23.8** | | **7.0** | **15** | **163.7 ± 0.9** | **0.074 ± 0.017** | **19.5 ± 6.0** |
| **AETC/DEA** |  | | **AETC** | **DEA** |  |  |  |  |  |
|  | **16** | **69.2** | **0** | **23.8** | **7.0** | **15** | **144.6 ± 0.4** | **0.102 ± 0.012** | **1.1 ± 0.1** |
|  | **17** | **69.2** | **5.95** | **17.85** | **7.0** | **15** | **119.6 ± 1.2** | **0.121 ± 0.027** | **21.2 ± 0.6** |
|  | **18** | **69.2** | **11.9** | **11.9** | **7.0** | **15** | **135.8 ± 3.4** | **0.219 ± 0.067** | **11.0 ± 0.9** |
|  | **19** | **69.2** | **17.85** | **5.95** | **7.0** | **15** | **172.8 ± 2.8** | **0.286 ± 0.018** | **8.5 ± 0.3** |

**Table S1.** Complete physicochemical characterisation of optimised nanogels synthesised with varying monomer contents. Data are presented as mean ± standard deviation for n = 3.

| **Reporter** | **Primer** | **Sequence (5' - 3')** |
| --- | --- | --- |
| β2 | Plasmid - Forward | AATTAAGAATTCTAATACGACTCACTATAGGGATGGG |
|  | Plasmid - Reverse | CTACAAGGACGACGATGACAAGTGAAGGCTCGAG |
| mCherry-Mango II RNA | Plasmid – Forward | AATTCTAATACGACTCACTATAGCGCCACCATGGTGAGCAAGGGCGAGGA |
|  | Plasmid – Reverse | CCCATTCGAACAAAAACTCATCTCAGAAGAGGATCTGTGAAATATGC |
| MII x9 | Sequence | TGTACAGCACGTACGAAGGAGAGGAGAGGAAGAGGAGAGTACGTGCTCTCGCACGTACGAAGGAGAGGAGAGGAAGAGGAGAGTACGTGCTCTCGCACGTACGAAGGAGAGGAGAGGAAGAGGAGAGTACGTGCTCTCCCTAGGGCACGTACGAAGGAGAGGAGAGGAAGAGGAGAGTACGTGCTCTCGCACGTACGAAGGAGAGGAGAGGAAGAGGAGAGTACGTGCTCTCGCACGTACGAAGGAGAGGAGAGGAAGAGGAGATACTACTCTCCCTAGG GTCGACGCACGTACGAAGGAGAGGAGAGGAAGAGGAGAGTACGTGCTCTCGCACGTACGAAGGAGAGGAGAGGAAGAGGAGAGTACGTGCTCTCGCACGTACGAAGGAGAGGAGAGGAAGAGGAGAGTACGTGCTCTCTTAATTAA |

**Table S2.** Primers used for synthesis of mRNA and templates.
